# Ingestion of a variety of non-animal-derived dietary protein sources results in diverse postprandial plasma amino acid responses which differ between young and older adults

**DOI:** 10.1017/S0007114524000163

**Published:** 2024-05-14

**Authors:** Ino van der Heijden, Sam West, Alistair J. Monteyne, Tim J. A. Finnigan, Doaa R. Abdelrahman, Andrew J. Murton, Francis B. Stephens, Benjamin T. Wall

**Affiliations:** 1 Department of Public Health and Sport Sciences, Faculty of Health and Life Sciences, Heavitree Road, University of Exeter, Exeter EX1 2LU, UK; 2 New Era Foods, Hutton Rudby, Yarm, UK; 3 Department of Surgery, University of Texas Medical Branch, Galveston, TX, USA; 4 Sealy Center on Aging, University of Texas Medical Branch, Galveston, TX, USA

**Keywords:** Plant protein, Algae, Mycoprotein, Amino acids, Bioavailability, Ageing

## Abstract

Whole-body tissue protein turnover is regulated, in part, by the postprandial rise in plasma amino acid concentrations, although minimal data exist on the amino acid response following non-animal-derived protein consumption. We hypothesised that the ingestion of novel plant- and algae-derived dietary protein sources would elicit divergent plasma amino acid responses when compared with vegan- and animal-derived control proteins. Twelve healthy young (male (m)/female (f): 6/6; age: 22 ± 1 years) and 10 healthy older (m/f: 5/5; age: 69 ± 2 years) adults participated in a randomised, double-blind, cross-over trial. During each visit, volunteers consumed 30 g of protein from milk, mycoprotein, pea, lupin, spirulina or chlorella. Repeated arterialised venous blood samples were collected at baseline and over a 5-h postprandial period to assess circulating amino acid, glucose and insulin concentrations. Protein ingestion increased plasma total and essential amino acid concentrations (*P* < 0·001), to differing degrees between sources (*P* < 0·001), and the increase was further modulated by age (*P* < 0·001). Postprandial maximal plasma total and essential amino acid concentrations were highest for pea (2828 ± 106 and 1480 ± 51 µmol·l^−1^) and spirulina (2809 ± 99 and 1455 ± 49 µmol·l^−1^) and lowest for chlorella (2053 ± 83 and 983 ± 35 µmol·l^−1^) (*P* < 0·001), but were not affected by age (*P* > 0·05). Postprandial total and essential amino acid availabilities were highest for pea, spirulina and mycoprotein and lowest for chlorella (all *P* < 0·05), but no effect of age was observed (*P* > 0·05). The ingestion of a variety of novel non-animal-derived dietary protein sources elicits divergent plasma amino acid responses, which are further modulated by age.

Dietary protein is vital to support whole-body tissue maintenance by providing amino acids acting as both signal and substrate for the stimulation and continuation of whole-body, and tissue-specific, protein synthesis^(1,2)^. For example, within skeletal muscle, the postprandial protein synthetic response is dictated by the rise and total availability of plasma (essential) amino acid (EAA) concentrations^(3–5)^, with leucine of particular interest^(6)^. The postprandial amino acid response is further modulated by various other factors, including protein digestibility, and the amino acid content and composition of the ingested protein source^(7,8)^. In addition, advancing age has been associated with a delayed or impaired protein digestibility and amino acid absorbability^(8,9)^ and/or an increased splanchnic extraction of amino acids^(10,11)^, reducing postprandial plasma amino acid availability.

The impact of ingesting different protein sources, and age, on postprandial amino acid responses has been extensively studied utilising animal-derived protein sources^(5,12–15)^, but increased production and consumption of animal protein is associated with growing environmental and ethical concerns^(16–18)^. Interest is growing in the potential utility of (more) sustainably produced, non-animal-derived protein sources which already comprise a large part of our habitual diet^(19)^. While non-animal-derived protein sources are typically considered of lesser anabolic quality than animal-derived proteins^(5,20,21)^, due to lower and less balanced EAA compositions, and/or inferior protein digestion and absorption kinetics^(22)^, few human studies investigating the *in vivo* aminoacidaemic response following non-animal-derived protein ingestion are available.

Proteins from legumes, such as lupin and pea, are commercially advanced and already used routinely within the food industry as supplemental proteins or food ingredients. Previous work has suggested lupin protein to be of lower nutritional quality, due to an unfavourable amino acid composition^(23)^, low Digestible Indispensable Amino Acid Score (DIAAS)^(24)^ and rodent data reporting low amino acid bioavailability^(25)^. On the other hand, despite being low in methionine, pea protein is high in other EAA and leucine^(17)^. Recent work reported equivalent *in vivo* ileal digestibility following pea and casein protein ingestion^(26)^ and robust post-exercise muscle protein synthetic responses following pea protein ingestion^(27)^. Aside from animal- and plant-derived proteins, other categories of proteins are generating scientific and commercial interest^(17,28)^. We recently identified fungal-derived mycoprotein as a high-quality protein-rich whole-food source, by demonstrating it is bioavailable upon ingestion^(29)^, robustly stimulates muscle protein synthesis (MPS) rates^(30–32)^ and, consequently, supports muscle adaptive responses during training^(33)^. A less commercially advanced alternative protein source is algae. Algae are protein- and EAA-rich, but typically used at present only as micronutrient-rich food supplements^(17)^, with the cyanobacterium spirulina (*Arthrospira platensis)* and microalgae chlorella (*Chlorella vulgaris)* widely available. Though human data assessing the impact of algae ingestion on plasma amino acid responses are lacking, animal studies have reported chlorella and spirulina ingestion to attenuate muscle atrophy and enhance MPS rates, respectively^(34,35)^.

In the present study, we undertook a scoping investigation into the impact of ingesting isonitrogenous boluses of a broad range of novel, promising dietary protein sources from plants (pea and lupin) and algae (spirulina and chlorella) on postprandial amino acid availability, and compared them with more thoroughly studied animal (milk) and non-animal (mycoprotein) controls in healthy young and older adults. We hypothesised that ingesting pea, lupin, spirulina and chlorella would elicit lower or equivalent plasma amino acid responses (i.e. either slower/lower peak concentrations or lower total postprandial amino acid availability) when compared with milk and mycoprotein and that the aminoacidaemic response would be lower in older compared with younger adults.

## Methods

### Participants

Twelve healthy young (age: 22 ± 1 years; BMI 22·1 ± 0·7 kg·m^−2^; male/female: 6/6) and 10 healthy older (69 ± 2 years; BMI 24·1 ± 0·7 kg·m^−2^; male/female: 5/5) adults volunteered to take part in this study (online Supplementary Fig. 1). Participant characteristics are displayed in Table 1. Exclusion criteria included a history of lactose intolerance or allergies to milk protein, mycoprotein/edible fungi/penicillin or algae foods; BMI below 18·5 or above 30·0 kg·m^−2^; high blood pressure (young adults, > 140/90 mmHg; older adults, > 150/90 mmHg); identifying as vegan; regular smokers or a current diagnosis of type 2 diabetes mellitus or CVD/complications. Participants were informed of the purpose of the study, experimental procedures and all potential risks prior to providing written informed consent. Older adults were excluded if they displayed high glycated haemoglobin (> 42 mmol·mol^−1^) or impaired liver function (alkaline phosphatase < 30 or > 130 iU·l^−1^, or alanine aminotransferase < 10 or > 33 iU·l^−1^, or total bilirubin > 21 µmol·l^−1^). The study was approved by the Sport and Health Sciences Ethics Committee of the University of Exeter (Ref No. 191023/A/09) in accordance with the latest version of the Declaration of Helsinki. The study was registered at ClinicalTrials.gov (NCT04297137). Recruitment and data collection were carried out in the Nutritional Physiology Research Unit at the University of Exeter between December 2019 and October 2022.

**Table 1.** Participants’ characteristics

	Young (*n* 12)		Older (*n* 10)		
	Means	sems	Means	sems	*P*-value
Sex (m/f)	6/6		5/5		n/a
Age (years)	22	1	69	2	< 0·001
Body mass (kg)	69	4	71	4	0·74
Height (cm)	176	2	171	3	0·16
BMI (kg·m^−2^)	22	1	24	1	0·08
Lean mass (kg)	57	4	52	4	0·38
Body fat (%)	18	2	27	2	0·01
Systolic BP (mmHg)	122	3	136	4	< 0·01
Diastolic BP (mmHg)	69	2	73	2	0·09
HbA_1c_ (mmol·mol^−1^)	n/a		38	1	n/a
ALT (IU·l^−1^)	n/a		19	3	n/a
ALP (IU·l^−1^)	n/a		76	9	n/a
Total bilirubin (µmol·l^−1^)	n/a		9	1	n/a

Values are means ± sems. ALP, alkaline phosphatase; ALT, alanine aminotransferase; BP, blood pressure; HbA_1c_, glycated haemoglobin.

### Pretesting

Prior to inclusion in the study, participants first completed a screening session which consisted of assessments of body mass, height, blood pressure, body composition (BodPod, Life Measurement, Inc.) and the completion of a routine medical screening questionnaire. A fasting blood sample was collected in the older population to assess circulating glycated haemoglobin, alkaline phosphatase, alanine aminotransferase and total bilirubin concentrations. Young participants were deemed healthy based on the response to the medical screening questionnaire and blood pressure. Older participants were deemed healthy based on the response to the medical screening questionnaire, blood pressure and the results of the fasting blood sample (Table 1).

### Study design

In a randomised, double-blind, cross-over design participants completed 6 experimental test days. Randomisation was performed by an independent person using a computerised randomiser. During each visit, participants ingested a beverage containing 30 g protein derived from milk (MILK), mycoprotein (MYCO), pea (PEA), lupin (LUP), spirulina (SPIR) or chlorella (CHLO). Arterialised venous blood samples were collected in the postabsorptive state and at regular time intervals over a 5-h postprandial period to assess circulating glucose, insulin and amino acid concentrations. Visual analogue scales were used at regular intervals to assess subjective ratings of appetite and beverage palatability. Test days were separated by at least 7 d to allow for complete digestion, absorption and metabolism of the test proteins and return to habitual dietary habits.

### Diet and physical activity

Participants were instructed to complete a 3-d food record to assess habitual dietary intake on 2 weekdays and 1 weekend day prior to taking part in the trial (data presented in Table 2). Participants were instructed to refrain from vigorous physical activity for 24 h before each test day and to cease taking any nutritional supplements during the study period. On the day before each test day, participants were instructed to keep their diet as consistent as possible and refrained from alcohol consumption. On the evening before each test day, participants were provided with a standardised meal. Participants were allowed to choose between 3 standardised meals, providing 44 %, 45 % or 50 % of energy as carbohydrate, 31 %, 29 % or 29 % of energy as fat, and 24 %, 23 % or 21 % of energy as protein, but then had to adhere to their choice prior to each experimental test day.

**Table 2.** Habitual dietary intake

	Young (*n* 12)		Older (*n* 10)		
	Means	sems	Means	sems	*P*-value
Energy (MJ·d^−1^)	9·8	0·7	8·4	0·7	0·17
Energy (kcal·d^−1^)	2336	166	2000	168	0·17
Protein (g·d^−1^)	97	10	75	4	0·06
Protein (g·kg^−1^ BM·d^−1^)	1·4	0·1	1·1	0·1	0·03
Protein (%En)	17	1	16	1	0·43
Carbohydrate (g·d^−1^)	254	23	242	28	0·73
Carbohydrate (%En)	45	1	51	2	0·22
Fat (g·d^−1^)	99	7	73	8	0·03
Fat (%En)	38	1	33	2	0·03
Fibre (g·d^−1^)	23	3	27	2	0·42

BM, body mass; En, energy.

Values are means ± sems. Data were analysed using an independent samples *t* test.

### Experimental procedures

**Fig. 1.**
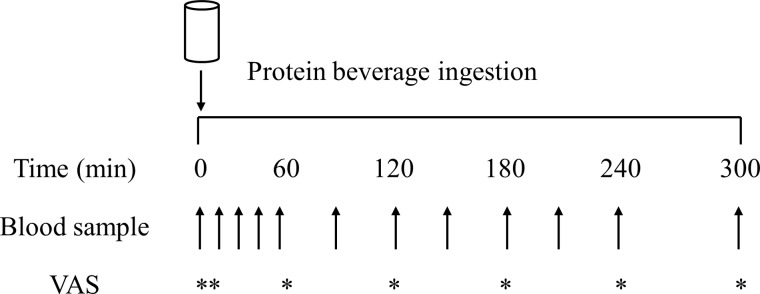
Schematic representation of the experimental protocol. VAS, visual analogue scale.

On all test days, participants reported to the laboratory at 08.00 h after a > 12 h overnight fast and were asked to rest in bed in a semi-supine position. The experimental protocol during each test day is shown in Fig. 1. A Teflon cannula was inserted into a dorsal vein of the hand and placed in a hot box (55°C) for arterialised venous blood sampling^(36)^. After 20 min, to allow complete venous blood arterialisation, a postabsorptive blood sample was collected and participants completed a visual analogue scale to assess subjective feelings of appetite. These 100 mm paper-based scales included questions regarding hunger, satisfaction, fullness, and prospective food consumption^(37)^. Afterwards, participants consumed one of the 6 protein containing beverages within 5 min (in a randomised and counterbalanced order) with completion indicating the start of the postprandial period (i.e. *t* = 0 min). Consumption of the beverage was followed by a 5 h postprandial period in which 11 blood samples were collected at *t* = 15, 30, 45, 60, 90, 120, 150, 180, 210, 240 and 300 min, while subjects remained in a semi-supine position throughout. Further appetite visual analogue scale recordings were collected at *t* = 5, 60, 120, 180, 240 and 300 min. A 100 mm paper-based scale assessing protein beverage palatability including questions regarding taste, aftertaste, texture and overall palatability was collected at *t* = 5 min. The distance between the point marked on the visual analogue scale and the left end of the scale was measured by the same researcher each time to minimise discrepancies, with collected data used to calculate individual and composite appetite and palatability scores as previously reported^(37)^.

### Experimental beverage preparations

Pea protein isolate (MyProtein™, THG plc, Manchester, UK), lupin (Indigo Herbs Ltd, Glastonbury, UK) spirulina (Bulk™, London, UK) and cracked-cell chlorella powders (Naturya Ltd, Bath, UK) were obtained from commercial suppliers. Freeze-dried mycoprotein was produced and obtained from Marlow Foods Ltd, Quorn Foods Stokesley, UK, as previously described^(38)^. Given milk is typically consumed as a nutrient-dense whole food, the more whole-food nature of the majority of the experimental protein sources and increasing appreciation of the role of non-protein aspects of the food matrix in regulating the postprandial amino acid and subsequent MPS responses^(39,40)^, the milk protein beverage was prepared to as closely represent a whole food as possible. The milk protein beverage consisted of commercially obtained instant full cream milk powder (Nestlé UK Ltd, York, UK) dissolved in skimmed milk (Tesco Stores Ltd, Waltham Cross, UK) to ensure isonitrogenous and iso-volumetric conditions could be achieved across beverages, while maintaining the milk protein to be considered as a ‘whole-food’ source of protein similar to mycoprotein. All protein sources were independently analysed (Premier Analytical Services) for energy, macronutrient and amino acid composition, with the details presented in Table 3. Based on incomplete amino acid profiling due to analytical issues, protein content was calculated as nitrogen (N) × 6·25 (N determined via the Kjeldahl method). While we concede the presence of non-protein nitrogen-containing factors potentially introduces error, we consider this to be minimal and in line with what is typically accepted within the food industry.

**Table 3.** Nutritional content of the protein containing test beverages

	MILK	MYCO	PEA	LUP	SPIR	CHLO
Macronutrients						
Protein (g)	30	30	30	30	30	30
Carbohydrate (g)	47·8	3·3	2·0	8·8	5·9	8·1
Fat (g)	16·1	4·2	2·5	5·9	1·3	3·5
Fibre (g)	< 0·1	13·5	< 0·1	19·9	4·3	3·9
Energy (kcal)	454	198	150	248	164	192
Energy (kJ)	1911	830	636	1035	692	810
Amino acid content (g)						
Alanine	1·0	1·7	1·3	1·1	2·4	2·5
Arginine	1·0	1·8	2·5	3·5	2·1	1·8
Aspartic acid	2·5	2·6	3·6	3·4	3·1	2·7
Glutamic acid	5·7	3·2	5·2	6·2	4·2	3·4
Glycine	0·6	1·2	1·2	1·3	1·5	1·7
Histidine	0·8	0·6	0·7	0·9	0·5	0·6
Isoleucine	1·6	1·2	1·3	1·3	1·7	1·0
Leucine	2·7	2·0	2·5	2·2	2·8	2·5
Lysine	2·6	2·0	2·2	1·5	1·5	2·4
Phenylalanine	1·5	1·2	1·6	1·2	1·4	1·4
Proline	2·9	1·2	1·2	1·2	1·1	1·5
Serine	1·8	1·3	1·6	1·6	1·6	1·2
Threonine	1·4	1·4	1·1	1·1	1·6	1·4
Tyrosine	1·2	0·9	1·1	1·2	1·3	1·0
Valine	1·9	1·4	1·5	1·2	1·9	1·7
EAA	12·6	9·8	10·9	9·4	11·4	11·0
NEAA	16·7	13·9	17·7	19·4	17·3	15·8
ΣAA	29·2	24·9	28·6	28·8	28·7	26·8

CHL, chlorella; EAA, essential amino acids; LUP, lupin; MILK, milk; MYC, mycoprotein; NEAA, non-essential amino acids; PEA, pea; SPIR, spirulina; ΣAA, sum of measured amino acids.

Protein content is calculated as nitrogen × 6·25.

Protein beverages were prepared the evening before test days by adding the amount of powder required to provide 30 g protein to 400 ml water, or 400 ml skimmed milk (MILK), and 50 ml of artificial energy-free vanilla flavouring (Jordan’s Skinny Mixes) and 25 ml of green energy-free food colouring (Tesco Stores Ltd.) for blinding purposes, making all drinks a dark green colour, and mixed for approximately 2 min using a food blender. Following drink consumption by the participant, an additional 100 ml was added to ‘rinse’ the bottle and ensure that all protein had been consumed, making a total fluid volume of 575 ml consumed by participants on each occasion. Double blinding of the drinks was achieved by having a different researcher to the one performing the experiment prepare the drinks in a metal, non-transparent bottle ready for consumption. Following ingestion of each drink, volunteers were asked to identify which condition they thought they had received which was noted down without feedback. Individual successful identification rates were as follows: MILK, 73 %, MYCO, 5 %, PEA, 5 %; LUP, 27 %; SPIR, 27 %; CHLO, 23 %, implying successful blinding with the exception of MILK.

### Blood sample analysis

Eight ml of arterialised venous blood was collected into a syringe at each sampling point. For each blood sample, a 20 µl plastic capillary was used and immediately analysed for blood glucose concentrations (Biosen C-Line GP+). A second part (∼4 ml) was collected in lithium heparin-containing tubes (BD vacutainer LH; BD Diagnostics) and centrifuged immediately at 4000 rpm at 4°C for 10 min to obtain blood plasma samples. The plasma supernatant was then removed, aliquoted and frozen at −80°C for subsequent analysis. The remaining blood was added to additional vacutainers (BD Vacutainer SST II tubes, BD Diagnostics) which were left to clot at room temperature for 30 min and then centrifuged at 4000 rpm at 4°C for 10 min to obtain blood serum. The serum supernatant was then removed, aliquoted and frozen at −80°C for subsequent analysis. Serum insulin concentrations were analysed using a commercially available assay kit (DRG Insulin ELISA, EIA-2935, DRG International Inc.). Plasma concentrations of phenylalanine, leucine, valine, isoleucine, lysine, histidine, glutamic acid, methionine, proline, serine, threonine, tyrosine, glycine and alanine were determined by GC-MS with electron impact ionisation (Agilent) as previously described^(32,41)^. Briefly, to prepare samples for GC-MS, 10 µl of 2 mM norleucine was added as an internal standard to 500 µl of plasma and deproteinised on ice with 500 µl of 15 % 5-sulfosalcylic acid. Samples were then vortexed and centrifuged at 4000 *g* for 10 min at 4°C. The supernatant was then loaded onto cation-exchange columns. Columns were filled with ddH_2_O, followed by 6 ml 0·5 M acetic acid and then washed 5 more times with ddH_2_O, with the columns allowed to drain between each step. The amino acids were then eluted with 2 ml of 6 M ammonium hydroxide (NH_4_OH). The eluate was dried using a Speed-Vac at 60°C and then derivatised via the addition of 50 μl of MTBSTFA + 1 % tert-butyldimethylchlorosilane and 50 μl of acetonitrile, followed by heating at 95°C for 45 min.

### Statistical analyses

Based on previous research using an identical cross-over design^(29)^, sample size was calculated with differences in postprandial plasma EAA incremental area under the curve (iAUC) between protein sources as the primary outcome measure. A sample size of 10 participants, including a 20 % dropout rate, was calculated with a power of 80 % and a significance level of 0·05 to detect a relevant difference of ∼15 % in EAA iAUC between protein sources. Based on previous studies displaying greater variance in the amino acid response to protein ingestion in young compared with older adults^(42–44)^, we opted to take a more conservative approach and selected a sample size of 12 young and 10 older adults. Secondary outcomes included temporal plasma amino acid and serum insulin responses, and the remaining responses were classified as tertiary. All postabsorptive and postprandial temporal plasma amino acid blood glucose and serum insulin concentrations are displayed in two separate graphs each, split for age to avoid graph congestion and improve visual appearance. However, all these measures were analysed within a single repeated-measures 3-way ANOVA test (one for each dependent variable) with age (young *v*. older) as a between-subject factor, and time and protein source (MILK *v*. MYCO *v*. PEA *v*. LUP *v*. SPIR *v*. CHLO) as within-subject factors. Subsequent 2-way ANOVAs were used to analyse time × protein source, time × age and protein source × age interactions. Total postprandial amino acid, blood glucose and serum insulin availabilities were calculated and represented as iAUC using the trapezoid rule. Differences in iAUCs as well as maximum concentrations (Cmax) and time to reach maximum concentrations (Tmax) were analysed with two-way ANOVAs with protein source and age as factors. For all ANOVAs, when significant interactions or main effects (when appropriate) were observed, Bonferroni *post hoc* tests were performed to locate individual differences. Data were tested for sphericity, and where violations occurred, the Greenhouse-Geisser correction was applied. Participant characteristics, dietary intake records and other differences between age groups, irrespective of protein source, were analysed using an independent samples *t* test with Welch correction where appropriate. Statistical significance was set at *P* < 0·05. All calculations were performed using SPSS Statistics version 28.0.0.0 (IBM Corp.), and all graphs were created using Graph Prism version 9.5.0. All data are expressed as means ± sems.

## Results

### Participants’ characteristics and habitual dietary intake

No differences in body mass, height, BMI or lean mass were found between young and older participants (all *P* > 0·05; Table 1). Body fat percentage and systolic blood pressure were higher in older compared with younger participants (both *P* < 0·05). Habitual diets did not differ between younger and older adults for energy, carbohydrate or fibre intakes (all *P* > 0·05; Table 2). Although absolute habitual protein intakes also did not differ between age groups (*P* = 0·06), relative protein intake expressed per kg body mass was higher in young (1·4 g/kg BM/d) compared with older participants (1·1 g/kg BM/d) (*P* = 0·03). Habitual fat intake was also higher in young compared with older participants (*P* = 0·03).

### Plasma amino acid concentrations

**Fig. 2.**
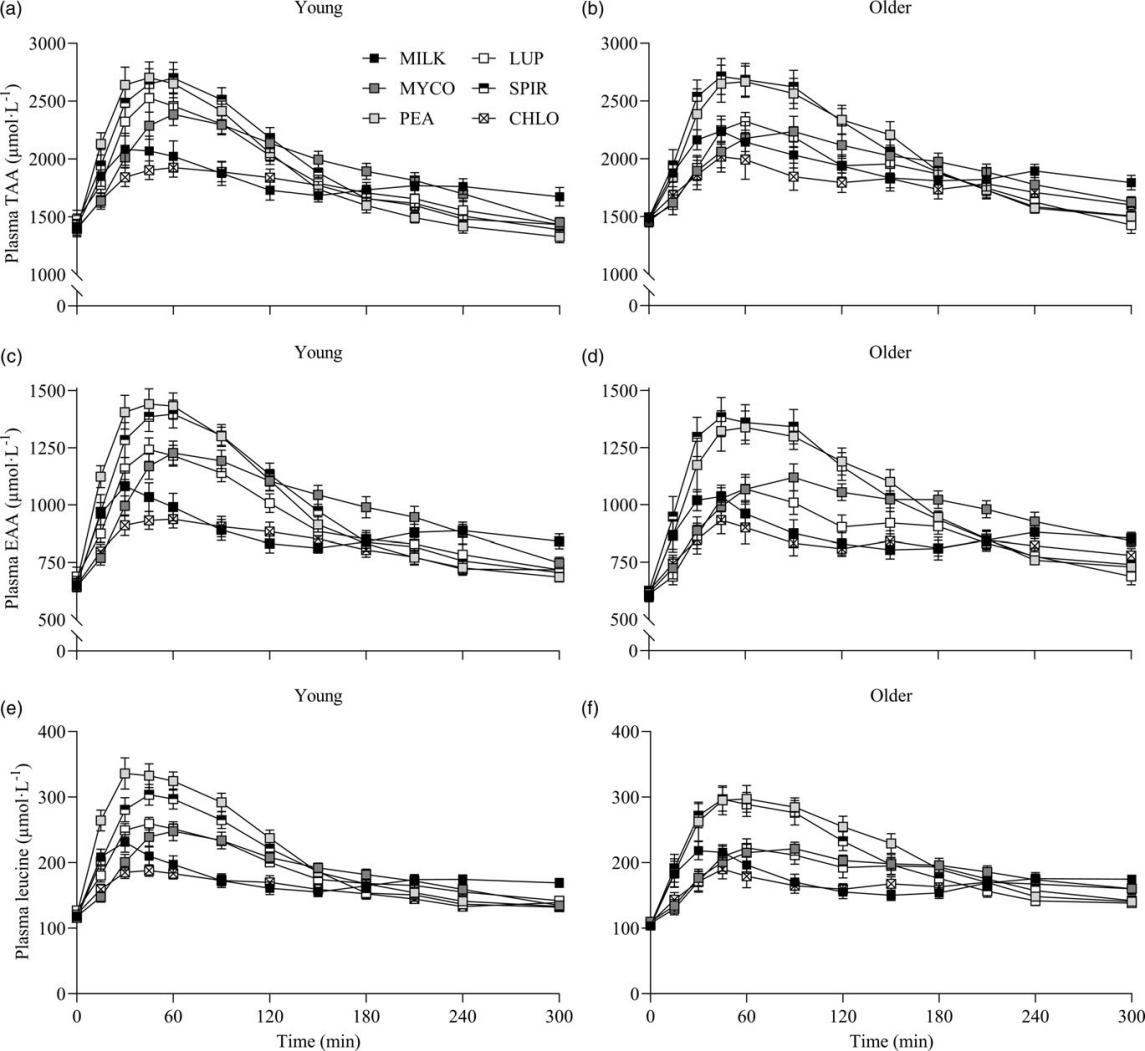
Plasma TAA (a), (b), EAA (c), (d) and leucine (e), (f) concentrations over time during the 5-h postprandial period following the ingestion of 30 g protein from milk, mycoprotein, pea, lupin, spirulina and chlorella in healthy young (*n* 12) and older (*n* 10) adults. Values are means ± sems. Plasma concentrations over time were analysed with 3-way repeated-measures ANOVA (time × protein × age), and outcomes are reported in online Supplementary Table 1. CHL, chlorella; EAA, essential amino acids; LUP, lupin; MILK, milk; MYC, mycoprotein; PEA, pea; SPIR, spirulina; TAA, total amino acids.

**Fig. 3.**
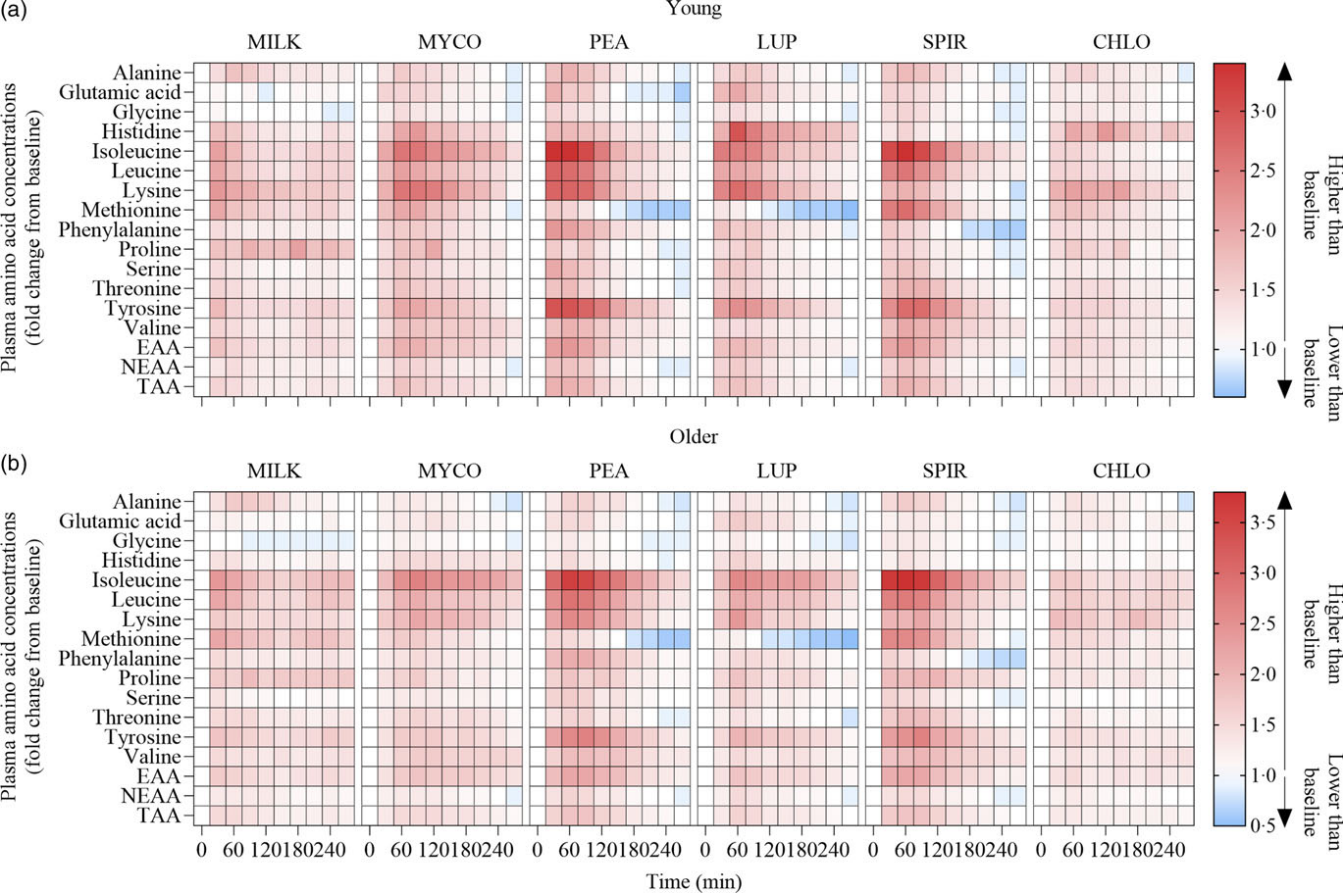
Plasma amino acid concentrations following the ingestion of 30 g protein from milk, mycoprotein, pea, lupin, spirulina and chlorella in healthy young (*n* 12) (a) and older (*n* 10) (b) adults displayed as a heatmap. The fold change from baseline (*t* = 0) has been determined for each time point. White, no changes in plasma amino acid concentrations when compared with baseline at the indicated time point; red, plasma amino acid concentrations are higher than baseline; blue, plasma amino acid concentrations are lower than baseline. CHL, chlorella; EAA, essential amino acids; LUP, lupin; MILK, milk; MYC, mycoprotein; PEA, pea; SPIR, spirulina; TAA, total amino acids.

The results of the statistical analyses (*P* values) concerning temporal changes in plasma total amino acid (TAA), essential amino acid (EAA), non-essential amino acid and individual amino acid concentrations following drink ingestion are reported in their entirety within online Supplementary Table 1 and the data are visualised in Figs. 2 & 3. All data for each parameter were tested within the same statistical test and figures are separated based on age for visual clarity only. From similar postabsorptive concentrations (all *P* > 0·05), plasma TAA, EAA and leucine concentrations increased following drink ingestion (time effects; all *P* < 0·001), but to a different extent between protein sources (time × protein interactions; all *P* < 0·001), and the increase was further modulated by age (time × protein × age interactions; all *P* < 0·05, respectively). For plasma EAA and leucine concentrations, a time × age interaction was also observed (both *P* < 0·05), though no main effect of age or protein × age interaction was observed (all *P* > 0·05).

**Fig. 4.**
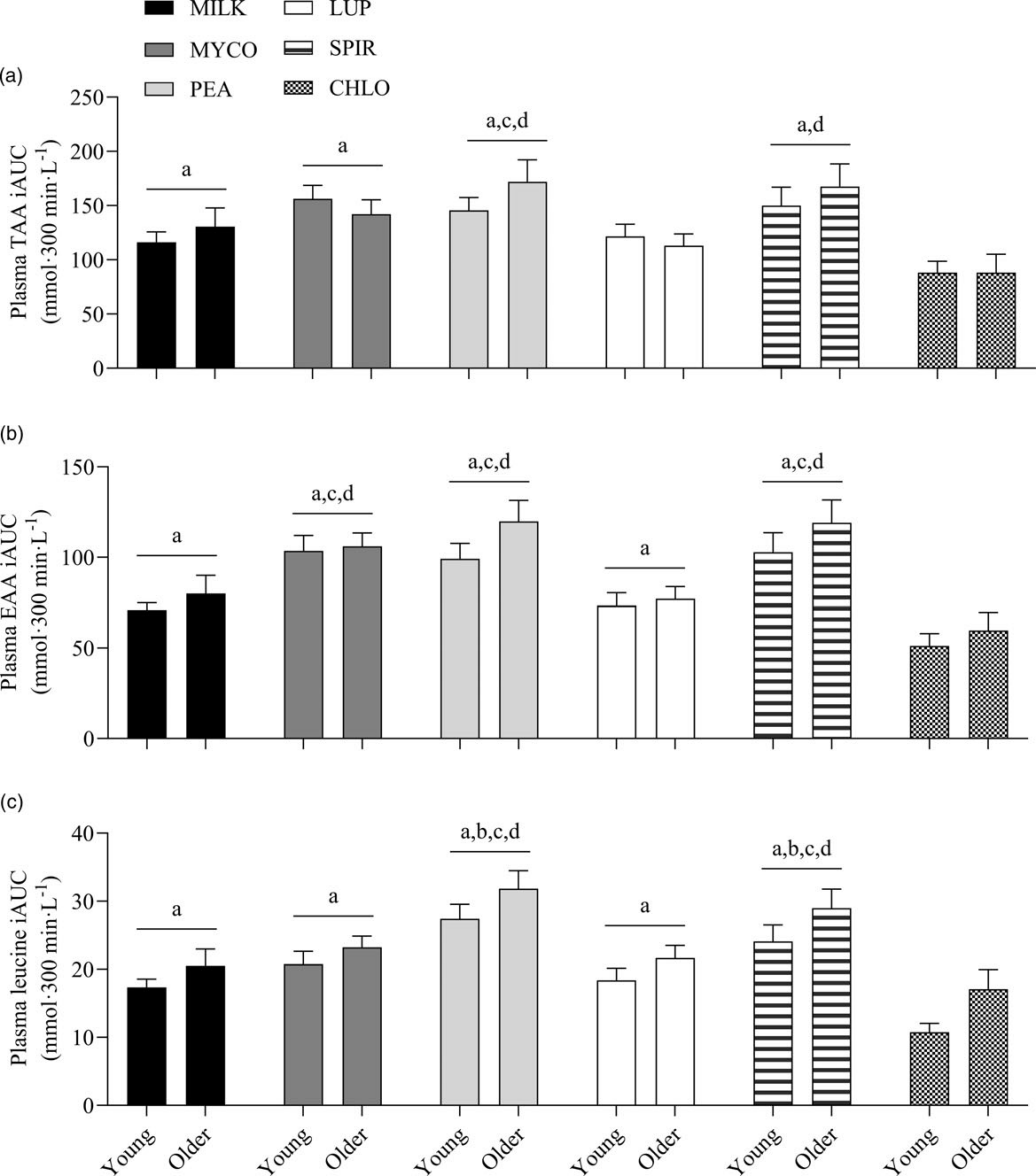
Total plasma TAA (a), EAA (b) and leucine (c) availabilities, expressed as iAUC, during the 5-h postprandial period following the ingestion of 30 g protein from milk, mycoprotein, pea, lupin, spirulina and chlorella in healthy young (*n* 12) and older (*n* 10) adults. Values are means ± sems. The iAUC data were analysed with 2-way ANOVA (protein × age), and outcomes are reported in online Supplementary Table 2. Data young and older adults are displayed in the same panels as no age or age or protein × age effects were observed (both *P* > 0·05) and Bonferroni *post hoc* tests were used to detect differences between protein sources (protein effect; *P* < 0·001). (a) significant difference to CHLO (*P* < 0·05); (b) significant difference to MYCO (*P* < 0·05); (c) significant different to MILK (*P* < 0·05); (d) significant difference to LUP (*P* < 0·05). CHL, chlorella; EAA, essential amino acids; iAUC, incremental area under the curve; LUP, lupin; MILK, milk; MYC, mycoprotein; PEA, pea; SPIR, spirulina; TAA, total amino acids.

Specifics of the statistical analyses for maximum plasma amino acid concentrations (Cmax), time to reach maximum concentrations (Tmax) and total plasma amino acid availabilities during the 5 h postprandial period (iAUC) following drink ingestion are reported in full in online Supplementary Table 2. Postprandial Cmax, Tmax and iAUC responses for TAA, EAA and leucine differed between protein sources (protein effects; all *P* < 0·01) but were not affected by age (age effects, protein × age interactions; all *P* > 0·05). Postprandial plasma TAA Cmax were highest, and similar, for PEA (2828 ± 106 µmol·l^−1^) and SPIR (2809 ± 99 µmol·l^−1^), lowest for CHLO (2054 ± 74 µmol·l^−1^) and MILK (2256 ± 77 µmol·l^−1^) (all *P* < 0·001), and were reached earliest for PEA (58 ± 5 min), LUP (57 ± 4 min), SPIR (57 ± 5 min) and MILK (52 ± 8 min) and latest for MYCO (100 ± 11 min) (all *P* < 0·05) (online Supplementary Table 3). Postprandial plasma EAA Cmax was highest for PEA (1481 ± 51 µmol·l^−1^) and SPIR (1455 ± 49 µmol·l^−1^), and lowest for CHLO (983 ± 35 µmol·l^−1^) (*P* < 0·001). However, EAA Tmax was lowest, and equivalent, for PEA (57 ± 5 min), LUP (64 ± 9 min), SPIR (57 ± 4 min) and MILK (48 ± 11 min) (*P* > 0·05) but faster than MYCO (108 ± 13 min) only (all *P* < 0·05) (online Supplementary Table 4). Postprandial plasma leucine Cmax was greater for PEA (342 ± 15 µmol·l^−1^) and SPIR (313 ± 11 µmol·l^−1^) *v*. LUP (255 ± 8 µmol·l^−1^), MYCO (249 ± 9 µmol·l^−1^) and MILK (241 ± 10 µmol·l^−1^), and lowest for CHLO (203 ± 8 µmol·l^−1^) (i.e. PEA, SPIR > LUP, MYCO, MILK > CHLO) (all *P* < 0·05), but maximum concentrations were reached earlier for SPIR (50 ± 4 min) and MILK (50 ± 14 min) *v*. MYCO (102 ± 13 min) only (all *P* < 0·05). Postprandial plasma TAA, EAA and leucine iAUCs are displayed in Fig. 4 and reported in online Supplementary Table 5. Plasma TAA and EAA iAUCs were highest, and equivalent, for PEA, SPIR and MYCO (*P* > 0·05) and lower in CHLO compared with all protein sources (all *P* < 0·01), except for TAA iAUC in LUP (*P* = 0·09). Plasma leucine iAUC was higher and similar in SPIR and PEA compared with other protein sources (all *P* < 0·01) and lower in CHLO compared with all protein sources (all *P* < 0·05).

Statistical analyses of remaining plasma amino acid responses in young and older adults are presented in online Supplementary Tables 1–5, and the data are visualised in Fig. 3. Plasma concentrations of all these amino acids increased following drink ingestion (time effects; all *P* < 0·001) and to differing degrees between protein sources (time × protein interactions; all *P* < 0·001). The increase was further modulated by age for plasma valine and isoleucine concentrations only (time × protein × age interactions; both *P* < 0·05), but main effects of age were observed for plasma glutamic acid, serine, threonine and tyrosine concentrations (age effects; all *P* < 0·05). In general, postprandial Cmax for remaining amino acids was highest following ingestion of PEA and SPIR and lowest for CHLO (all *P* < 0·05), and, apart from lysine, glutamic acid, threonine and tyrosine, no age or protein × age effects on Cmax were observed (all *P* > 0·05). Tmax differed between protein sources (protein effects; all *P* < 0·05), except for alanine and tyrosine (both *P* > 0·05), and no effects of age were observed except for isoleucine (age effect; *P* = 0·04, protein × age interaction; *P* = 0·03) and non-essential amino acid (protein × age interaction; *P* = 0·04). Plasma amino acid iAUCs were different between protein sources for remaining amino acids (protein effects; all *P* < 0·001), except for histidine, glutamic acid and non-essential amino acid concentrations (all *P* > 0·05), and no protein × age interactions were observed (all *P* > 0·05). Postprandial amino acid availabilities were positive for all amino acids except for methionine following ingestion of LUP and for glycine in MILK.

**Fig. 5.**
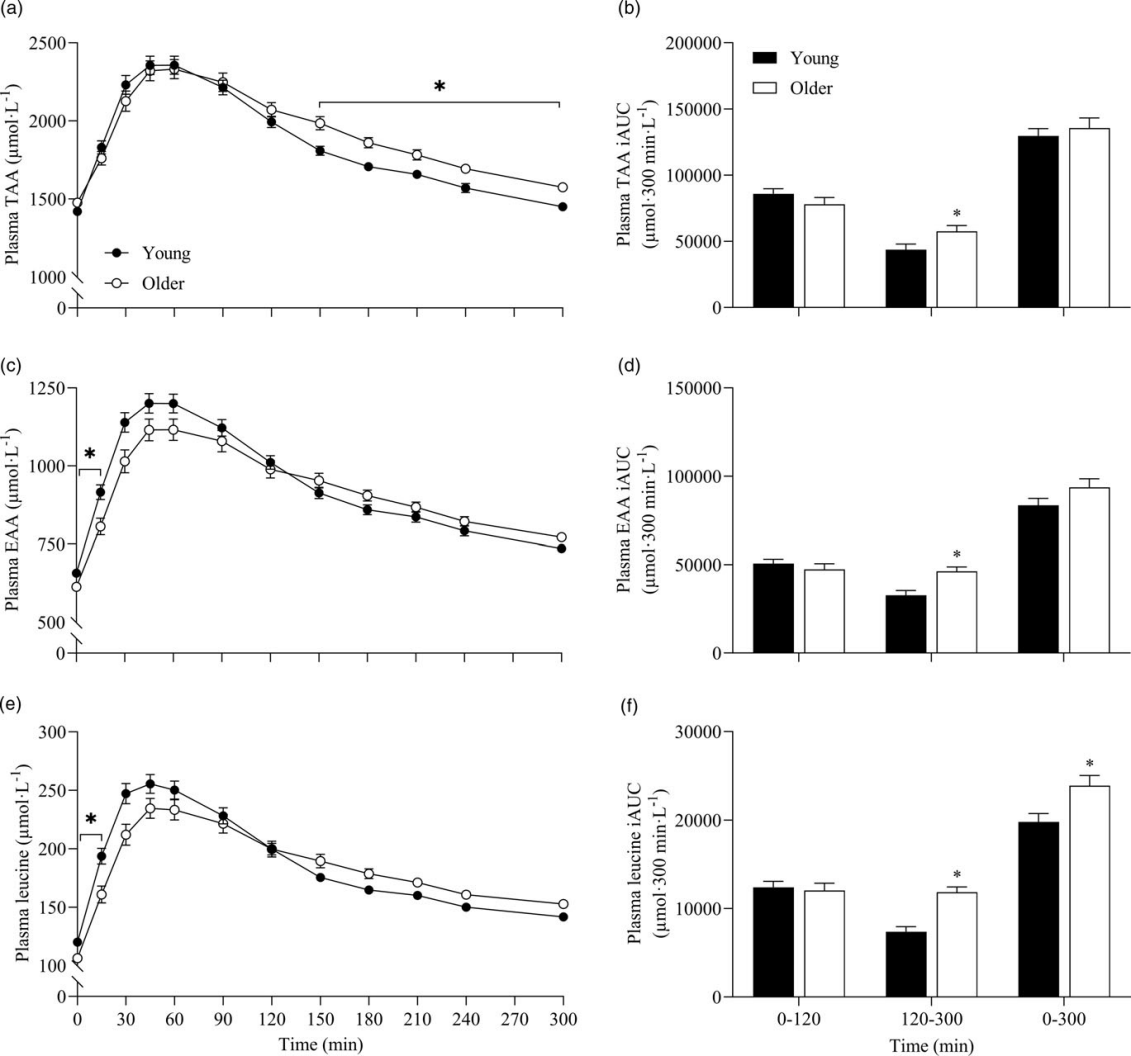
Combined data, irrespective of protein source, showing plasma TAA (a), EAA (c) and leucine (e) concentrations over time and total TAA (b), EAA (d) and leucine (f) responses, expressed as iAUC, during the early (0–120 min), late (120–300 min) and total (0–300 min) postprandial period following the ingestion of 30 g protein from milk, mycoprotein, pea, lupin, spirulina and chlorella in healthy young (*n* 12) and older (*n* 10) adults. Values are means ± sems. Plasma concentrations over time were analysed with 2-way repeated-measures ANOVA (time × group), with Bonferroni *post hoc* tests applied to detect individual differences. iAUCs were analysed with independent samples *t* test. Time × group interactions; all *P* < 0·001. *, significant difference between groups (*P* < 0·05). EAA, essential amino acids; iAUC, incremental area under the curve; TAA, total amino acids.

To further assess the effects between our older and younger cohorts on the postprandial plasma amino acid responses, we collapsed data across protein sources (thereby removing the independent variable of protein source and creating 6 ‘replicates’ per participant that were equivalent across populations) and performed additional statistical analyses (Fig. 5). This approach showed that postprandial TAA, EAA and leucine concentrations increased following drink ingestion (time effects; all *P* < 0·001) to differing degrees between young and older participants (time × age interactions; all *P* < 0·001). Plasma TAA, EAA and leucine Cmax were similar between young and older participants, but plasma EAA and leucine concentrations increased more rapidly (Tmax; both *P* < 0·05) and were higher from *t* = 0–15 min (all *P* < 0·05) in young compared with older participants. Conversely, postprandial plasma TAA concentrations were lower from *t* = 150–300 min in young compared with older participants (*P* < 0·05). Consequently, leucine iAUC over the 5 h postprandial period, as well as TAA, EAA and leucine iAUCs in the late (120–300 min), but not early (0–120 min), phase of the postprandial periods were greater in older compared with younger participants (all *P* < 0·05).

### Blood glucose and serum insulin concentrations

Statistical analysis details for blood glucose and serum insulin responses are reported in online Supplementary Tables 1–5. Blood glucose concentrations increased following drink ingestion (time effect; *P* < 0·001), but to differing degrees between protein sources (time × protein interaction; both *P* < 0·001) (online Supplementary Fig. 2). The increase in blood glucose concentrations was further modulated by age (time × protein × age interaction; *P* = 0·004), and a main effect of age was also observed (*P* = 0·02). Blood glucose Cmax was highest for MILK in young and older participants (protein effects; *P* < 0·001) and was higher in older compared with young (age, protein × age effects; *P* < 0·05). However, no differences in blood glucose iAUC between young and older participants were observed (age, protein × age effects; both *P* > 0·05).

**Fig. 6.**
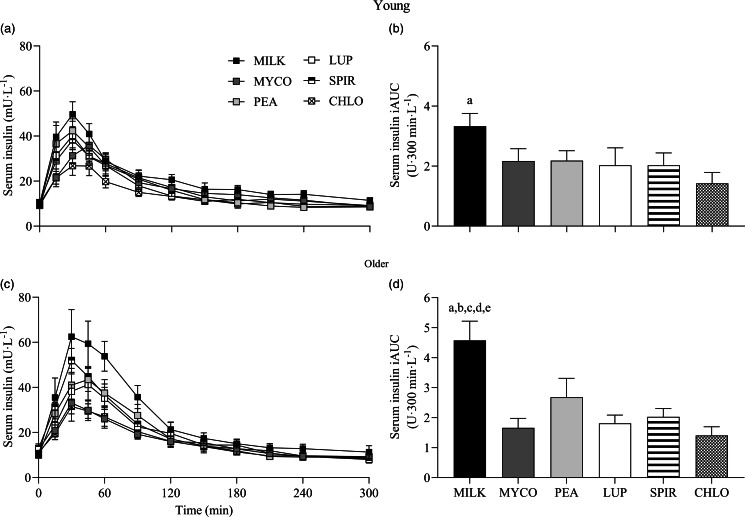
Serum insulin concentrations over time (a), (c) and total insulin availabilities (b), (d), expressed as iAUC, during the 5-h postprandial period following the ingestion of 30 g protein from milk, mycoprotein, pea, lupin, spirulina and chlorella in healthy young (*n* 12) and older (*n* 10) adults. Values are means ± sems. Serum insulin concentrations over time were analysed with 3-way repeated-measures ANOVA (time × protein × age), and outcomes are reported in online Supplementary Table 1. Serum insulin iAUC was analysed with 2-way ANOVA (protein × age), and outcomes are reported in online Supplementary Table 2. For iAUC data, panels were separated for young and older adults as a protein × age effect (*P* = 0·019) was observed, and Bonferroni *post hoc* tests were used to detect differences between protein sources (protein effects; both *P* < 0·001). (a), significant difference to CHLO (*P* < 0·05); (b), significant difference to MYCO (*P* < 0·05); (c), significant different to LUP (*P* < 0·05); (d), significant difference to PEA (*P* < 0·05); (e), significant difference to SPIR (*P* < 0·05). CHL, chlorella; iAUC, incremental area under the curve; LUP, lupin; MILK, milk; MYC, mycoprotein; PEA, pea; SPIR, spirulina.

Serum insulin responses over time following drink ingestion are displayed in Fig. 6(a) and (c) and separated for age for visual clarification (statistical outcomes are reported in online Supplementary Table 1). Serum insulin concentrations increased following drink ingestion (time effect; *P* < 0·001) and to a different extent between protein sources (time × protein interaction; *P* < 0·001). The increase was further modulated by age (time × protein × age interaction; *P* = 0·026). Postprandial serum insulin Cmax was highest and lowest for MILK and CHLO (protein effect; *P* < 0·001), respectively, in both young (MILK, 53 ± 5; CHLO, 31 ± 4 mU·l^−1^) and older participants (MILK, 72 ± 10; CHLO, 35 ± 4 mU·l^−1^), but Cmax was affected by age (protein × age interaction; *P* = 0·02). Serum insulin iAUC was highest for MILK in young and older participants (protein effect; *P* < 0·001), but a protein × age interaction was observed (*P* = 0·019). Serum iAUC responses for young and older participants are displayed in separate panels in Fig. 6(b) and (d).

### Visual analogue scale responses

Statistical analyses for subjective ratings of hunger, satisfaction, fullness, prospective food intake and composite appetite score during the experimental trials for young and older participants are presented in online Supplementary Table 6. Following drink ingestion, main effects of time for all appetite variables were observed (all *P* < 0·001) but this was neither affected by protein source or age (time × protein, time × age, time × protein × age interactions; all *P* > 0·05). Responses for individual variables and composite appetite score are displayed in online Supplementary Fig. 3. Composite appetite score decreased following drink ingestion and returned to a postabsorptive score at *t* = 120 min, before trending to be greater at the end of the postprandial period (*P* = 0·07). Main effects of age were observed for hunger and composite appetite (both *P* < 0·01), indicating average scores were higher in young (44 ± 4 and 53 ± 3 mm, respectively) compared with older (26 ± 4 and 41 ± 3 mm, respectively) participants.

Palatability scores for taste, aftertaste, texture and overall palatability in young and older participants are presented in online Supplementary Table 7. Scores differed between protein sources (protein effect; all *P* < 0·001) and were modulated by age for texture only (protein × age interaction; *P* = 0·003). In general, across all variables, scores were highest for MILK and PEA and lowest for MYCO, SPIR and CHLO.

## Discussion

In the present study, we assessed the systemic amino acid, glucose and insulin responses to the ingestion of isonitrogenous boluses of novel plant- (pea and lupin) and algae- (spirulina and chlorella) derived dietary protein sources compared with more established animal- (milk) and vegan- (mycoprotein) derived comparators, in healthy young and older adults. Plasma amino acid concentrations increased most rapidly following pea and spirulina ingestion resulting in an equivalent total postprandial amino acid availability as mycoprotein. Lupin and milk protein ingestion resulted in lower total postprandial amino acid availabilities, and slower and lower responses still were observed following chlorella ingestion. Despite similar plasma amino acid concentrations being observed across protein sources between young and older adults, the generic postprandial aminoacidaemic response appeared to be delayed by advancing age.

Whole body and tissue (especially skeletal muscle) protein synthetic responses to dietary protein ingestion have been extensively studied utilising animal-derived proteins (e.g.^(5,12–15,45)^). This body of work has shown that various crucial aspects are to be considered, including the (essential) amino acid composition of a protein source^(3,4)^, the postprandial rise in circulating insulin concentrations^(46,47)^, as well as the postprandial peak concentration (Tmax), time to reach peak concentration (Cmax) and total availability (iAUC) of circulating EAAs (particularly leucine) once ingested^(5,6,45,48)^, though the ultimate anabolic response is complex and driven by the interplay between these factors^(49)^. Bearing these variables in mind, such studies have established what constitutes a ‘high-quality’ dietary protein, with obvious examples in work examining milk proteins^(50–52),^ and, more recently, we identified fungal-derived mycoprotein as a similarly high-quality non-animal-derived protein^(30–33)^. In the present study, and in line with our hypothesis, we replicate our previous findings by demonstrating mycoprotein represents a bioavailable protein source by eliciting a relatively slow but sustained postprandial increase in systemic amino acid and insulin concentrations. However, contrary to our hypothesis, and not in line with our previous work^(29,30)^, milk ingestion resulted in a ∼19 % lower postprandial total amino acid availability than mycoprotein, likely attributed to the manner in which the milk protein was provided. Given milk is typically consumed as a nutrient-dense food and recent work demonstrating a robust MPS response following whole-food ingestion^(14,30,32,40,53,54)^, we provided a whole food as opposed to an isolated milk protein beverage (as we had in previous work). However, this resulted in higher carbohydrate and fat contents in the milk drink (Table 3), which likely dampened the plasma aminoacidaemic response, as these macronutrients have been demonstrated to delay gastric emptying and/or protein digestion and amino acid absorption^(43,55,56)^. Accordingly, this presumably elicited a slower (i.e. Tmax) and lower (i.e. Cmax) increase in postprandial plasma amino acid concentrations in comparison with a typical response expected following isolated milk protein ingestion. In addition, the highest plasma glucose and serum insulin responses were unsurprisingly observed following milk protein ingestion, attributable to the higher carbohydrate and energy contents. This may have dampened the postprandial amino acid response by increasing whole-body rates of amino acid disappearance (i.e. greater uptake by peripheral tissue) and/or decreasing endogenous amino acid appearance into the circulation due to reduced tissue protein breakdown, effects which would have remained unseen in this work due to measuring plasma concentrations alone. However, our data are in agreement with previous work demonstrating the impact of the milk matrix on postprandial amino acid availability^(57,58)^ and are not necessarily reflective of the subsequent MPS response and the anabolic potential of the provided milk protein beverage^(51,57)^.

With respect to our plant-based proteins, the ingestion of pea protein resulted in a rapid increase in circulating insulin and amino acid concentrations, while lupin ingestion elicited slower and lower responses. This resulted in a ∼37 % lower EAA availability for lupin compared with pea, presumably attributable to an impaired rate of appearance (i.e. absorbability) given an increased clearance under less insulinemic conditions is unlikely. While this effect may be driven by the higher amino acid content in the pea drink provided (Table 3), it is unlikely to be exclusively explanatory given the marked disconnect we observed between EAA content and postprandial plasma responses across protein sources. For example, pea protein contained 1·1 g (∼11 %) more and 1·7 g (∼14 %) fewer EAAs compared with mycoprotein and milk, respectively; but total postprandial EAA availability upon pea ingestion was ∼4 % and ∼37 % higher than mycoprotein and milk, respectively. Being commercially advanced as a food ingredient and widely available as a (sport) nutrition supplement, we provided pea as a protein isolate (i.e. protein content > 80 % of total mass). This likely explains the observed rapid increase in postprandial circulating insulin and amino acid concentrations, a finding consistent with recent studies^(26,27)^, and suggests pea protein to be an ideal source for stimulating tissue anabolism. In contrast, the aminoacidaemic response to lupin ingestion may have been delayed, or impaired, due to the high fibre content within its whole-food matrix (Table 3), which has been proposed to modulate the rise in postprandial amino acid concentrations^(29,30)^. Moreover, lupin, in comparison with other legumes, is rich in phytic acid and oligosaccharides^(59)^, so-called ‘antinutritional factors’ suggested to impair plant protein digestion and amino acid absorption^(60)^. Removal of such ingredients for the production of protein isolates enhances digestibility and amino acid absorbability^(61)^. Accordingly, ingesting isolated lupin protein may elicit a different aminoacidaemic response that is (more) similar to isolated pea protein. However, to our knowledge, lupin protein isolates are not (yet) available for commercial purposes, and, therefore, the present data imply pea protein isolate may be a more favourable source (compared with ‘whole lupin’) for supporting muscle protein anabolism. A final point worth considering is that legumes are typically low in methionine according to WHO/FAO/UNU guidelines^(62)^. Our data demonstrate low postprandial methionine availabilities following ingestion of both pea and lupin, an observation also observed previously^(25,63)^. This is of relevance since single *in vivo* amino acid deficiencies are proposed to provide substrate limitation to the continuation of postprandial MPS rates^(64)^, though we did not observe this in a recent study^(27)^.

A rarely considered alternative dietary protein source is algae, remarkably EAA-rich, with no obvious amino acid deficiencies^(62)^. Despite being relatively well-matched for macronutrient and amino acid content (Table 3), we observed markedly divergent postprandial systemic insulin and amino acid responses upon spirulina compared with chlorella ingestion. While spirulina evoked a rapid increase in plasma leucine and EAA concentrations, greater than that of both control conditions, chlorella appeared to be digested slower and to an overall lower extent. This translated to a ∼60 % greater postprandial EAA availability for spirulina compared with chlorella and the lowest EAA and leucine (and most other amino acids) availabilities for chlorella across all protein sources. Moreover, spirulina ingestion provoked the highest maximum plasma leucine and EAA concentrations (equivalent with pea) whereas chlorella was inferior across all comparator proteins. The discrepancy between algal species likely relates to the individual food matrices and cellular structures. Spirulina (technically a cyanobacterium) consists of helix-shaped cells, without discrete cell walls^(65)^, whereas chlorella species contain a complex multi-layered cell wall consisting of various insoluble and indigestible polysaccharides (predominantly glucosamine and cellulose)^(66)^ which exist to provide structural stability and rigidity to the cell^(67)^. Despite using commercially available ‘cracked-cell’ chlorella, the present data imply that current industry practices may not be fully effective for this purpose, and further optimisation to enhance human digestibility and *in vivo* nutrient (including amino acid) availability would be advantageous.

As opposed to alterations in protein digestibility and/or amino acid absorbability, divergent plasma aminoacidaemic responses across protein sources may also reflect changes in whole-body amino acid kinetics (i.e. rates of appearance and disappearance), mediated by differences in the hormonal milieu provoked by the macronutrient composition of the drinks. Though we cannot confirm this in the present study, it is worth noting that divergent insulin responses are not necessarily reflective of differences in postprandial amino acid availability and/or whole-body protein net balance^(43,56)^, with a moderate rise (up to ∼30 mU·l^−1^) in circulating insulin concentrations considered sufficient to maximise tissue anabolism^(68)^. Given we observed a similar pattern, albeit to differing magnitudes, in postprandial insulinaemia (and an increase to > 30 mU·l^−1^) across protein sources (Fig. 6), we consider the influence of altered whole-body kinetics on postprandial aminoacidaemia to be less than that compared with aspects of protein digestion and absorption.

Advancing age is associated with a reduced anabolic sensitivity to dietary protein ingestion, at least within skeletal muscle^(69,70)^, making it relevant to study how age may influence the metabolism of alternative dietary proteins. We observed that postprandial amino acid and insulin responses to the various protein sources were broadly similar between the two age cohorts, but some subtle (but consistent) differences across protein sources allowed us to collapse all the data to assess the effects across groups (Fig. 5). This approach revealed the postprandial increase in leucine and EAA concentrations to be modestly slower, but not lower, in older compared with younger participants. This observation was striking given we matched absolute protein doses across cohorts, so this effect occurred despite the likelihood of a smaller plasma distribution volume in the older cohort^(42,44)^ (albeit our body composition data reveal minimal differences between groups). Hard conclusions here are difficult as we were unable to directly assess whole-body amino acid kinetics and/or metabolic fate due to the absence of either intravenously or exogenously administered isotopically labelled amino acids. Nevertheless, we speculate that these findings are consistent with previous more comprehensive reports demonstrating the exogenous rate of appearance of amino acids derived from intrinsically labelled dietary proteins was attenuated in older compared with younger adults^(43–45,71)^. This is likely attributable to impaired or delayed protein digestion and amino acid absorption^(8,9)^, increased amino acid retention by splanchnic tissues such as the gut and/or altered first-pass liver metabolism^(10,11)^. While our data only provide circulating amino acid concentrations, and not metabolic fluxes, it is interesting to note that, in contrast to the slower response in older volunteers, we observed *higher* plasma TAA, EAA and leucine availabilities in the later postprandial phase (2–5 h) in our older cohort. These data imply that, in addition to altered digestion and absorption, lower (or at least delayed) rates of amino acid disappearance from the circulation into peripheral tissues, including (and likely the largest contributor) skeletal muscle, occurred in older adults. Indeed, this is broadly in line with a body of evidence showing a blunted anabolic response to protein ingestion at the site of the muscle in older adults, irrespective of hyperaminoacidemia^(21,72)^. Consequently, the present findings reiterate likely age-related perturbations in postprandial dietary protein handling which, coupled with a greater satiation response from a protein-rich meal (online Supplementary Table 6; in line with^(73)^), collectively emphasise the challenge of developing age-specific dietary protein recommendations to support healthy ageing, especially when considering alternative protein sources (which were generally less palatable than more ‘established’ animal-derived protein (online Supplementary Table 7)).

In conclusion, ingestion of 30 g of protein derived from animal- plant-, fungal- and algal-derived sources elicits markedly different postprandial plasma amino acid responses which implies future work should address what impact this has on whole-body and/or muscle protein metabolic responses to consuming alternative dietary proteins. Ageing is accompanied by a delayed postprandial systemic plasma amino acid response emphasising the consideration of protein source on tissue protein remodelling with advancing age. Our data imply pea and spirulina to be high-quality dietary protein sources that should join mycoprotein and milk as obvious candidates for future study in this space.

## Supporting information

van der Heijden et al. supplementary material 1 van der Heijden et al. supplementary material

van der Heijden et al. supplementary material 2 van der Heijden et al. supplementary material

van der Heijden et al. supplementary material 3 van der Heijden et al. supplementary material

van der Heijden et al. supplementary material 4 van der Heijden et al. supplementary material

## Supplementary material

For supplementary material/s referred to in this article, please visit https://doi.org/10.1017/S0007114524000163
