# Supplementary material for: Ingestion of a variety of non-animal-derived dietary protein sources results in diverse postprandial plasma amino acid responses which differ between young and older adults
Source: Br J Nutr. 2024 Jan 15;131(9):1540–53. doi: 10.1017/S0007114524000163 (PMC11043913; doi:10.1017/S0007114524000163)
Supplement: van der Heijden et al. supplementary material 1 — van der Heijden et al. supplementary material [file S0007114524000163sup001.docx]

**Ingestion of a variety of non-animal-derived dietary protein sources results in diverse postprandial plasma amino acid responses which differ between young and older adults**

by Ino van der Heijden, Sam West, Alistair J. Monteyne, Tim J.A. Finnigan, Doaa R. Abdelrahman, Andrew J. Murton, Francis B. Stephens, and Benjamin T. Wall.

Online Supplemental Data

**Supplemental Table 1.** Statistical analyses of temporal changes in plasma amino acid, blood glucose and serum insulin concentrations following drink ingestion.

|  | Time | Protein | Age | Time × protein | Time × age | Protein × age | Time × protein × age |
| --- | --- | --- | --- | --- | --- | --- | --- |
| Alanine | *P*<0.001 | *P*<0.001 | *P*=0.995 | *P*<0.001 | *P*=0.513 | *P*=0.933 | *P*=0.137 |
| Glutamic acid | *P*<0.001 | *P*=0.731 | *P*=0.036 | *P*<0.001 | *P*=0.145 | *P*=0.399 | *P*=0.328 |
| Glycine | *P*<0.001 | *P*<0.001 | *P*=0.278 | *P*<0.001 | *P*=0.376 | *P*=0.711 | *P*=0.212 |
| Histidine | *P*<0.001 | *P*<0.001 | *P*=0.387 | *P*<0.001 | *P*=0.722 | *P*=0.362 | *P*=0.688 |
| Isoleucine | *P*<0.001 | *P*<0.001 | *P*=0.548 | *P*<0.001 | *P*=0.055 | *P*=0.110 | *P*=0.006 |
| Leucine | *P*<0.001 | *P*<0.001 | *P*=0.506 | *P*<0.001 | *P*=0.016 | *P*=0.338 | *P*=0.017 |
| Lysine | *P*<0.001 | *P*<0.001 | *P*=0.052 | *P*<0.001 | *P*=0.475 | *P*=0.005 | *P*=0.094 |
| Methionine | *P*<0.001 | *P*<0.001 | *P*=0.808 | *P*<0.001 | *P*=0.618 | *P*=0.351 | *P*=0.650 |
| Phenylalanine | *P*<0.001 | *P*<0.001 | *P*=0.934 | *P*<0.001 | *P*=0.107 | *P*=0.590 | *P*=0.105 |
| Proline | *P*<0.001 | *P*<0.001 | *P*=0.070 | *P*<0.001 | *P*=0.173 | *P*=0.020 | *P*=0.262 |
| Serine | *P*<0.001 | *P*<0.001 | *P*=0.039 | *P*<0.001 | *P*=0.077 | *P*=0.455 | *P*=0.081 |
| Threonine | *P*<0.001 | *P*<0.001 | *P*=0.001 | *P*<0.001 | *P*=0.010 | *P*=0.563 | *P*=0.259 |
| Tyrosine | *P*<0.001 | *P*<0.001 | *P*=0.025 | *P*<0.001 | *P*=0.149 | *P*=0.221 | *P*=0.217 |
| Valine | *P*<0.001 | *P*<0.001 | *P*=0.178 | *P*<0.001 | *P*=0.014 | *P*=0.410 | *P*=0.028 |
| EAA | *P*<0.001 | *P*<0.001 | *P*=0.430 | *P*<0.001 | *P*=0.043 | *P*=0.255 | *P*=0.028 |
| NEAA | *P*<0.001 | *P*=0.084 | *P*=0.143 | *P*<0.001 | *P*=0.340 | *P*=0.445 | *P*=0.088 |
| TAA | *P*<0.001 | *P*<0.001 | *P*=0.481 | *P*<0.001 | *P*=0.129 | *P*=0.415 | *P*=0.035 |
|  |  |  |  |  |  |  |  |
| Glucose | *P*<0.001 | *P*<0.001 | *P*=0.020 | *P*<0.001 | *P*<0.001 | *P*=0.020 | *P*=0.026 |
| Insulin | *P*<0.001 | *P*<0.001 | *P*=0.487 | *P*<0.001 | *P*=0.162 | *P*=0.020 | *P*=0.004 |

Main (time, protein, age) and interaction (time × protein, time × age, protein × age, time × protein × age) effects from 3-way ANOVAs used to analyse temporal changes in plasma amino acid, blood glucose and serum insulin concentrations during the 5 h postprandial period following the ingestion of 30 g protein from milk, mycoprotein, pea, lupin, spirulina, and chlorella ingestion in healthy young (*n*=12) and older adults (*n*=10). EAA, essential amino acids; NEAA, non-essential amino acid; TAA, total amino acids.

**Supplemental Table 2.** Statistical analyses of plasma amino acid, blood glucose and serum insulin maximum concentrations (Cmax), time to reach maximum concentrations (Tmax), and total availabilities during the 5 h postprandial period (iAUC) following drink ingestion.

|  |  | Cmax |  |  | Tmax |  |  | iAUC |  |
| --- | --- | --- | --- | --- | --- | --- | --- | --- | --- |
|  | Protein | Age | Protein × age | Protein | Age | Protein × age | Protein | Age | Protein × age |
| Alanine | *P*<0.001 | *P*=0.923 | *P*=0.282 | *P*=0.188 | *P*=0.885 | *P*=0.172 | *P*<0.001 | *P*=0.240 | *P*=0.366 |
| Glutamic acid | *P*<0.001 | *P*=0.040 | *P*=0.228 | *P*<0.001 | *P*=0.340 | *P*=0.840 | *P*=0.210 | *P*=0.912 | *P*=0.432 |
| Glycine | *P*<0.001 | *P*=0.405 | *P*=0.498 | *P*<0.001 | *P*=0.074 | *P*=0.293 | *P*<0.001 | *P*=0.056 | *P*=0.523 |
| Histidine | *P*<0.001 | *P*=0.500 | *P*=0.440 | *P*=0.026 | *P*=0.656 | *P*=0.238 | *P*=0.063 | *P*=0.963 | *P*=0.233 |
| Isoleucine | *P*<0.001 | *P*=0.808 | *P*=0.150 | *P*<0.001 | *P*=0.041 | *P*=0.031 | *P*<0.001 | *P*=0.031 | *P*=0.501 |
| Leucine | *P*<0.001 | *P*=0.412 | *P*=0.104 | *P*=0.002 | *P*=0.056 | *P*=0.360 | *P*<0.001 | *P*=0.107 | *P*=0.738 |
| Lysine | *P*<0.001 | *P*=0.123 | *P*=0.022 | *P*=0.004 | *P*=0.423 | *P*=0.787 | *P*<0.001 | *P*=0.069 | *P*=0.086 |
| Methionine | *P*<0.001 | *P*=0.654 | *P*=0.353 | *P*=0.027 | *P*=0.159 | *P*=0.480 | *P*<0.001 | *P*=0.942 | *P*=0.409 |
| Phenylalanine | *P*<0.001 | *P*=0.489 | *P*=0.926 | *P*=0.022 | *P*=0.197 | *P*=0.370 | *P*<0.001 | *P*=0.566 | *P*=0.510 |
| Proline | *P*<0.001 | *P*=0.174 | *P*=0.011 | *P*<0.001 | *P*=0.743 | *P*=0.706 | *P*<0.001 | *P*=0.009 | *P*=0.124 |
| Serine | *P*<0.001 | *P*=0.058 | *P*=0.629 | *P*=0.002 | *P*=0.517 | *P*=0.343 | *P*<0.001 | *P*=0.111 | *P*=0.298 |
| Threonine | *P*<0.001 | *P*=0.002 | *P*=0.648 | *P*=0.037 | *P*=0.347 | *P*=0.163 | *P*<0.001 | *P*=0.145 | *P*=0.715 |
| Tyrosine | *P*<0.001 | *P*=0.081 | *P*=0.220 | *P*=0.016 | *P*=0.056 | *P*=0.668 | *P*<0.001 | *P*=0.049 | *P*=0.281 |
| Valine | *P*<0.001 | *P*=0.197 | *P*=0.078 | *P*<0.001 | *P*=0.117 | *P*=0.508 | *P*<0.001 | *P*=0.504 | *P*=0.902 |
| EAA | *P*<0.001 | *P*=0.440 | *P*=0.277 | *P*<0.001 | *P*=0.165 | *P*=0.334 | *P*<0.001 | *P*=0.313 | *P*=0.651 |
| NEAA | *P*<0.001 | *P*=0.331 | *P*=0.201 | *P*<0.027 | *P*=0.651 | *P*=0.036 | *P*=0.090 | *P*=0.574 | *P*=0.234 |
| TAA | *P*<0.001 | *P*=0.829 | *P*=0.276 | *P*<0.001 | *P*=0.272 | *P*=0.828 | *P*<0.001 | *P*=0.708 | *P*=0.304 |
|  |  |  |  |  |  |  |  |  |  |
| Glucose | *P*<0.001 | *P*=0.018 | *P*=0.004 | *P*=0.758 | *P*=0.763 | *P*=0.668 | *P*<0.001 | *P*=0.972 | *P*=0.608 |
| Insulin | *P*<0.001 | *P*=0.555 | *P*=0.023 | *P*=0.011 | *P*=0.147 | *P*=0.820 | *P*<0.001 | *P*=0.747 | *P*=0.019 |

Main (protein, age) and interaction (protein × age) effects from 2-way ANOVAs used to analyse plasma amino acid, blood glucose and serum insulin peak concentrations, time to reach peak concentrations, and total availabilities during the 5 h postprandial period following the ingestion of 30 g protein from milk, mycoprotein, pea, lupin, spirulina, and chlorella ingestion in healthy young (*n*=12) and older adults (*n*=10). Cmax, maximum concentration; EAA, essential amino acids; iAUC, incremental area under the curve; NEAA, non-essential amino acid; TAA, total amino acids; Tmax, time to reach maximum concentrations.

**Supplemental Table 3.** Maximum plasma amino acid, blood glucose and serum insulin concentrations following drink ingestion. Data are separated between young and older adults when a significant effect of age (*P*<0.05), or a protein × age interaction (*P*<0.05) was observed, as reported in **Supplemental Table 2**.

|  | | MILK | MYCO | PEA | LUP | SPIR | CHLO | *P*-value |
| --- | --- | --- | --- | --- | --- | --- | --- | --- |
| Alanine | | 474±24^b^ | 395±20 | 424±19 | 426±18 | 485±26^a,b^ | 387±27 | <0.001 |
| Glutamic acid | Young | 152±23 | 212±29 | 224±30^a^ | 217±27^a,c^ | 199±26 | 160±17 | <0.001 |
| Glutamic acid | Older | 262±29 | 243±23 | 284±21 | 274±29 | 258±26 | 232±21 | 0.459 |
| Glycine | | 199±9 | 234±8^c^ | 246±12^c^ | 246±9^c^ | 257±13^c^ | 232±14 | <0.001 |
| Histidine | | 126±2^a^ | 114±10 | 133±11^a,b,f^ | 137±11^a,f^ | 112±9 | 107±11 | <0.001 |
| Isoleucine | | 194±10^a^ | 229±11^a,b^ | 300±16^a,b,c,d^ | 235±9^a,b^ | 310±15^a,b,c,d^ | 148±8 | <0.001 |
| Leucine | | 241±10^a^ | 249±9^a^ | 342±15^a,b,c,d^ | 255±8^a^ | 313±11^a,b,c,d^ | 203±8 | <0.001 |
| Lysine | Young | 56±6 | 74±5^a,c,f^ | 76±7^a,c,f^ | 64±6^a^ | 56±5 | 49±5 | <0.001 |
| Lysine | Older | 71±6 | 69±5 | 97±7^a,b,c,e,f^ | 70±5 | 71±2 | 60±5 | <0.001 |
| Methionine | | 31±1^a,b,d,e^ | 24±1^d^ | 22±1^d^ | 18±1 | 42±1^a,b,c,d,e^ | 23±1^d^ | <0.001 |
| Phenylalanine | | 68±3^a^ | 68±2 | 90±3^a,b,c,d,f^ | 62±6 | 72±3^a^ | 60±2 | <0.001 |
| Proline | Young | 96±15 | 95±12 | 92±18 | 100±15 | 79±17 | 70±10 | 0.109 |
| Proline | Older | 151±17^a,d^ | 87±15 | 133±20 | 104±17 | 103±12 | 105±12 | 0.001 |
| Serine | | 200±9 | 219±10^a^ | 268±13^a,b,c^ | 243±10^a,c^ | 251±14^a,c^ | 187±9 | <0.001 |
| Threonine | Young | 167±9 | 202±10^a^ | 190±12 | 191±16 | 231±17^a,c^ | 162±10 | <0.001 |
| Threonine | Older | 124±7 | 152±14 | 149±14 | 124±6 | 179±20 | 125±13 | 0.001 |
| Tyrosine | Young | 67±7 | 69±7^a^ | 91±8^a,b,c^ | 80±6^a,c^ | 97±7^a,b,c^ | 49±4 | <0.001 |
| Tyrosine | Older | 81±6 | 76±6 | 110±9^a,b,c^ | 88±7^a^ | 125±12^a,b,c,d^ | 67±5 | <0.001 |
| Valine | | 281±8 | 341±13^a,c,d^ | 366±12^a,c,d^ | 291±8 | 367±14^a,c,d^ | 278±11 | <0.001 |
| EAA | | 1116±36^a^ | 1247±36^a^ | 1480±51^a,b,c,d^ | 1205±36^a^ | 1455±49^a,b,c,d^ | 983±35 | <0.001 |
| NEAA | | 1180±52 | 1194±46 | 1352±64^a^ | 1303±47^a^ | 1361±57^a^ | 1086±53 | <0.001 |
| TAA | | 2256±77 | 2420±74^a^ | 2828±106^a,b,c,d^ | 2500±74^a,c^ | 2809±99^a,b,c^ | 2053±83 | <0.001 |
|  | |  |  |  |  |  |  |  |
| Glucose | Young | 5.8±0.2^a,b,d,e^ | 5.1±0.1 | 5.1±0.1 | 5.1±0.1 | 5.2±0.1^a^ | 5.0±0.1 | <0.001 |
| Glucose | Older | 6.9±0.3^a,b,d,e,f^ | 5.4±0.2 | 5.3±0.1 | 5.5±0.1 | 5.4±0.2 | 5.4±0.2 | <0.001 |
| Insulin | Young | 53±5^a^ | 38±4 | 51±7^a^ | 44±8 | 44±5^a^ | 31±4 | 0.003 |
| Insulin | Older | 72±10^a,b,d,e^ | 38±8 | 47±6 | 45±7 | 53±3^a,b^ | 35±4 | <0.001 |

Maximum plasma amino acid (µmol·L^-1^), blood glucose (mmol·L^-1^) and serum insulin (mU·L^-1^) concentrations following the ingestion of 30 g protein from milk, mycoprotein, pea, lupin, spirulina, and chlorella ingestion in healthy young (*n*=12) and older adults (*n*=10). Values are means±SEMs. Data were analysed with 2-way ANOVA (protein × age interaction), as reported in **Supplemental Table 2**, and *P*-value represent main effect of protein. In case a main effect of protein was observed, Bonferroni *post hoc* tests were applied to detect individual differences. a, significant difference to CHLO (*P*<0.05); b, significant difference to MYCO (*P*<0.05); c, significant difference to MILK (*P*<0.05); d, significant difference to LUP (*P*<0.05); e, significant difference to PEA (*P*<0.05); f, significant difference to SPIR (*P*<0.05). CHL, chlorella; EAA, essential amino acids; LUP, lupin; MILK, milk; MYC, mycoprotein; NEAA, non-essential amino acids; PEA, pea; SPIR, spirulina; TAA, total amino acids.

**Supplemental Table 4.** Time to reach peak plasma amino acid, serum insulin and blood glucose concentrations following drink ingestion. Data are separated between young and older adults when a significant effect of age (*P*<0.05), or a protein × age interaction (*P*<0.05) was observed, as reported in **Supplemental Table 2**.

|  | | MILK | MYCO | PEA | LUP | SPIR | CHLO | *P*-value |
| --- | --- | --- | --- | --- | --- | --- | --- | --- |
| Alanine | | 74±8 | 69±6 | 63±6 | 72±8 | 60±5 | 80±8 | 0.188 |
| Glutamic acid | | 125±18 | 106±10 | 51±4^b,c^ | 57±4^b,c^ | 69±11 | 82±11 | <0.001 |
| Glycine | | 55±12^a^ | 91±11 | 54±5^a^ | 55±5^a^ | 58±6^a,b^ | 125±15 | <0.001 |
| Histidine | | 97±18 | 91±14 | 57±7 | 68±9 | 69±7 | 110±15 | 0.026 |
| Isoleucine | Young | 31±3 | 91±17 | 56±8 | 48±5 | 63±8 | 50±10 | 0.021 |
| Isoleucine | Older | 63±27 | 131±20 | 62±9 | 111±22 | 50±7 | 101±28 | 0.003 |
| Leucine | | 50±14^b^ | 102±13 | 58±6^b^ | 70±11 | 50±4^b^ | 89±17 | 0.002 |
| Lysine | | 84±16 | 98±10 | 61±5^b^ | 53±5^b^ | 60±5^b^ | 83±11 | 0.004 |
| Methionine | | 59±16 | 82±9 | 40±4^b^ | 42±2^b^ | 48±4^b^ | 64±8 | 0.027 |
| Phenylalanine | | 65±15 | 87±10 | 57±6 | 59±10 | 39±3^b^ | 80±15 | 0.022 |
| Proline | | 132±16 | 76±9^c^ | 55±9^c^ | 77±14 | 74±9 | 76±12 | <0.001 |
| Serine | | 44±11^b^ | 95±13 | 48±4^b^ | 50±3^b^ | 48±4 | 83±15 | 0.002 |
| Threonine | | 79±14 | 109±15 | 63±6 | 66±8 | 70±8 | 83±11 | 0.037 |
| Tyrosine | | 73±19 | 93±11 | 60±6 | 78±11 | 60±5 | 91±15 | 0.160 |
| Valine | | 115±23 | 132±13 | 70±6^a,b^ | 75±11^a,b^ | 72±6^a,b^ | 142±17 | <0.001 |
| EAA | | 48±10^b^ | 108±13 | 57±5^b^ | 64±9^b^ | 57±4^b^ | 87±13 | <0.001 |
| NEAA | Young | 91±19 | 63±9 | 51±6^a^ | 49±2^a^ | 54±5^a^ | 94±13 | 0.025 |
| NEAA | Older | 69±11 | 92±16 | 62±8 | 81±15 | 57±8 | 68±5 | 0.249 |
| TAA | | 53±8 | 100±11 | 58±5^b^ | 57±4^b^ | 57±5^a,b^ | 85±10 | <0.001 |
|  | |  |  |  |  |  |  |  |
| Glucose | | 48±12 | 50±10 | 40±4 | 36±6 | 36±6 | 40±3 | 0.758 |
| Insulin | | 36±3 | 49±4 | 35±3^b^ | 38±4^b^ | 38±3 | 40±3 | 0.011 |

Time to reach maximum (min) plasma amino acid, blood glucose and serum insulin concentrations following the ingestion of 30 g protein from milk, mycoprotein, pea, lupin, spirulina, and chlorella ingestion in healthy young (*n*=12) and older adults (*n*=10). Values are means±SEMs. Data were analysed with 2-way ANOVA (protein × age interaction), as reported in **Supplemental Table 2**, and *P*-value represent main effect of protein. In case a main effect of protein was observed, Bonferroni *post hoc* tests were applied to detect individual differences. a, significant difference to CHLO (*P*<0.05); b, significant difference to MYCO (*P*<0.05); c, significant difference to MILK (*P*<0.05). CHL, chlorella; EAA, essential amino acids; LUP, lupin; MILK, milk; MYC, mycoprotein; NEAA, non-essential amino acids; PEA, pea; SPIR, spirulina; TAA, total amino acids.

**Supplemental Table 5**. Postprandial total plasma amino acid, serum insulin and blood glucose availability, expressed as iAUC, during the 5 h postprandial period following drink ingestion. Data are separated between young and older adults when a significant effect of age (*P*<0.05), or a protein × age interaction (*P*<0.05) was observed, as reported in **Supplemental Table 2**.

|  | | MILK | MYCO | PEA | LUP | SPIR | CHLO | *P*-value |
| --- | --- | --- | --- | --- | --- | --- | --- | --- |
| Alanine | | 25.0±3.2^a,b,d^ | 12.5±2.4 | 16.1±2.5 | 10.8±2.5 | 17.2±3.2 | 12.4±2.5 | <0.001 |
| Glutamic acid | | 3.2±1.3 | 2.9±2.4 | 6.1±1.9 | 8.2±1.6 | 4.2±2.2 | 4.9±1.4 | 0.210 |
| Glycine | | -2.3±1.1 | 5.1±0.9^c^ | 3.9±1.1^c^ | 3.3±1.3^c^ | 4.9±1.3^c^ | 4.9±1.1^c^ | <0.001 |
| Histidine | | 5.4±1.0 | 6.2±1.2 | 4.3±1.8 | 6.6±1.1 | 1.7±0.9 | 1.7±0.9 | 0.063 |
| Isoleucine | Young | 14.1±1.2^a^ | 24.5±1.7^a,c^ | 26.1±1.6^a,c,d^ | 21.3±1.6^a,c^ | 27.7±1.8^a,c,d^ | 5.6±0.7 | <0.001 |
| Isoleucine | Older | 17.9±2.0^a,c^ | 28.1±2.4^a,c^ | 32.9±2.8^a,c,d^ | 25.0±1.9^a,c^ | 35.2±3.3^a,b,c,d^ | 10.8±1.7 | <0.001 |
| Leucine | | 18.8±1.3^a^ | 21.9±1.3^a^ | 29.4±1.7^a,b,c,d^ | 19.9±1.3^a^ | 26.3±1.9^a,b,c,d^ | 13.6±1.6 | <0.001 |
| Lysine | | 4.7±0.5 | 6.4±0.5^a,f^ | 6.1±0.6^d,f^ | 3.9±0.5 | 3.2±0.5 | 3.7±0.6 | <0.001 |
| Methionine | | 2.5±0.2^a,b,d,e^ | 1.5±0.1^d,e^ | 0.0±0.1^d^ | -0.7±0.1 | 2.9±0.3^a,b,d,e^ | 1.2±0.2^d,e^ | <0.001 |
| Phenylalanine | | 3.2±0.4^f^ | 3.7±0.3^f^ | 6.1±0.5^a,b,c,d,f^ | 3.5±0.3^f^ | 0.6±0.4 | 2.8±0.4^f^ | <0.001 |
| Proline | Young | 8.1±1.8 | 4.2±1.1 | 1.0±0.7 | 2.7±1.2 | 1.1±1.0 | 3.0±0.9 | <0.001 |
| Proline | Older | 13.6±2.9^a,b^ | 3.4±0.7 | 6.3±1.8 | 5.7±1.4 | 6.6±2.3 | 4.2±1.4 | 0.001 |
| Serine | | 5.9±0.9 | 10.4±1.2^a,c^ | 11.0±1.0^a,c^ | 10.0±1.0^a,c^ | 9.0±1.2^a^ | 4.3±1.0 | <0.001 |
| Threonine | | 7.1±0.9 | 10.6±1.6 | 6.3±0.8 | 5.0±1.3 | 12.6±1.5^a,c,d,e^ | 6.2±1.1 | <0.001 |
| Tyrosine | Young | 4.1±0.5^a^ | 4.5±0.6 | 7.1±0.7^a,c^ | 5.8±0.7^a^ | 7.2±0.9^a^ | 2.3±0.5 | <0.001 |
| Tyrosine | Older | 6.7±1.3^a^ | 5.8±0.7 | 10.1±1.5^a,b,c,d^ | 7.1±0.8^a^ | 10.8±1.8^a,b,c^ | 3.9±0.9 | <0.001 |
| Valine | | 17.5±1.3 | 28.4±1.9^a,c,d^ | 27.1±2.0^a,c,d^ | 13.9±1.3 | 31.9±2.3^a,c,d^ | 15.9±1.8 | <0.001 |
| EAA | | 75.0±5.0^a^ | 104.7±5.6^a,c,d^ | 108.6±7.2^a,c,d^ | 75.0±4.9^a^ | 110.2±8.2^a,c,d^ | 55.0±5.7 | <0.001 |
| NEAA | | 47.6±5.3 | 45.0±4.9 | 48.9±5.0 | 42.7±4.3 | 47.8±6.3 | 33.2±5.0 | 0.090 |
| TAA | | 122.7±9.4^a^ | 149.7±9.0^a^ | 157.5±11.4^a,c,d^ | 117.7±7.7 | 158.0±13.1^a,d^ | 88.2±9.3 | <0.001 |
|  | |  |  |  |  |  |  |  |
| Glucose | | 60.3±17.0^a,b,e,f^ | -1.1±14.8 | -26.3±13.6 | -10.1±16.8 | -20.7±10.1 | -16.1±12.8 | <0.001 |
| Insulin | Young | 3.3±0.4^a^ | 2.2±0.4 | 2.2±0.3 | 2.0±0.6 | 2.0±0.4 | 1.4±0.4 | <0.001 |
| Insulin | Older | 4.6±0.6^a,b,d,e,f^ | 1.7±0.3 | 2.7±0.6 | 1.8±0.3 | 2.0±0.3 | 1.4±0.3 | <0.001 |

Total postprandial plasma amino acid (mmol·300 min·L^-1^), blood glucose (mmol·300 min·L^-1^) and serum insulin (U·300 min·L^-1^) availabilities over the 5 h postprandial period, expressed as iAUC, following the ingestion of 30 g protein from milk, mycoprotein, pea, lupin, spirulina, and chlorella ingestion in healthy young (*n*=12) and older adults (*n*=10). Values are means±SEMs. Data were analysed with 2-way ANOVA (protein × age interaction), as reported in **Supplemental Table 2**, and *P*-value represent main effect of protein. In case a main effect of protein was observed, Bonferroni *post hoc* tests were applied to detect individual differences. a, significant difference to CHLO (*P*<0.05); b, significant difference to MYCO (*P*<0.05); c, significant difference to MILK (*P*<0.05); d, significant difference to LUP (*P*<0.05); e, significant difference to PEA (*P*<0.05); f, significant difference to SPIR (*P*<0.05). CHL, chlorella; EAA, essential amino acids; LUP, lupin; MILK, milk; MYC, mycoprotein; NEAA, non-essential amino acids; PEA, pea; SPIR, spirulina; TAA, total amino acids.

**Supplemental Table 6.** Statistical analyses of temporal changes in subjective appetite ratings following drink ingestion.

|  | Time | Protein | Age | Time × protein | Time × age | Protein × age | Time × protein × age |
| --- | --- | --- | --- | --- | --- | --- | --- |
| Hunger | *P*<0.001 | *P*=0.381 | *P*=0.008 | *P*=0.130 | *P*=0.787 | *P*=0.375 | *P*=0.508 |
| Satisfaction | *P*<0.001 | *P*=0.066 | *P*=0.373 | *P*=0.365 | *P*=0.375 | *P*=0.175 | *P*=0.119 |
| Fullness | *P*<0.001 | *P*=0.736 | *P*=0.682 | *P*=0.243 | *P*=0.264 | *P*=0.054 | *P*=0.301 |
| Prospective food intake | *P*<0.001 | *P*=0.634 | *P*=0.057 | *P*=0.246 | *P*=0.954 | *P*=0.202 | *P*=0.675 |
| Composite appetite | *P*<0.001 | *P*=0.390 | *P*=0.017 | *P*=0.357 | *P*=0.780 | *P*=0.195 | *P*=0.594 |

Main (time, protein, age) and interaction (time × protein, time × age, protein × age, time × protein × age) effects from 3-way ANOVAs used to analyse temporal changes in subjective appetite ratings during the 5 h postprandial period following the ingestion of 30 g protein from milk, mycoprotein, pea, lupin, spirulina, and chlorella ingestion in healthy young (*n*=12) and older adults (*n*=10).

**Supplemental Table 7.** Subjective protein beverage palatability responses.

|  |  | MILK | MYCO | PEA | LUP | SPIR | CHLO | *P*-value | | |
| --- | --- | --- | --- | --- | --- | --- | --- | --- | --- | --- |
|  |  |  |  |  |  |  |  | Protein | Age | Protein × age |
| Taste | | 61±5^a,b,c,e^ | 36±5^a^ | 44±5^a^ | 33±5 | 28±4 | 16±3 | <0.001 | 0.958 | 0.375 |
| Aftertaste | | 59±6^a,b,c,e^ | 36±5 | 45±5^a^ | 29±5 | 29±4 | 23±4 | <0.001 | 0.465 | 0.434 |
| Texture | Young | 75±7^a,b,c,d,e^ | 24±5 | 46±6^b^ | 58±6^b^ | 41±7 | 45±5 | <0.001 | 0.491 | 0.003 |
|  | Older | 58±9^a,c^ | 39±7 | 55±7 | 36±8 | 39±6 | 35±7 | <0.001 |  |  |
| Overall palatability | | 65±5^a,b,c,e^ | 31±4 | 47±5^a,b^ | 34±5 | 28±4 | 20±3 | <0.001 | 0.822 | 0.075 |

Subjective hunger, aftertaste, texture and overall palatability scores (mm) obtained 5 min following the ingestion of 30 g protein from milk, mycoprotein, pea, lupin, spirulina, and chlorella ingestion in healthy young (*n*=12) and older adults (*n*=10). Values are means±SEMs. Data were analysed with 2-way ANOVA (protein × age interaction). In case an interaction effect was observed, data were shown separately for young and older participants. In case a main effect of protein was observed, Bonferroni *post hoc* tests were applied to detect individual differences. a, significant difference to CHLO (*P*<0.05); b, significant difference to MYCO (*P*<0.05); c, significant difference to LUP (*P*<0.05); d, significant difference to PEA (*P*<0.05); e, significant difference to SPIR. CHL, chlorella; LUP, lupin; MILK, milk; MYC, mycoprotein; PEA, pea; SPIR, spirulina.

**Supplemental Figure legends**

**Supplemental Figure 1.** Participant flow diagram. CHL, chlorella; LUP, lupin; MILK, milk; MYC, mycoprotein; PEA, pea; SPIR, spirulina.

**Supplemental Figure 2.** Blood glucose concentrations over time (A, B) and total glucose availability (C), expressed as iAUC, during the 5 h postprandial period following the ingestion of 30 g protein from milk, mycoprotein, pea, lupin, spirulina, and chlorella in healthy young (*n*=12) and older (*n*=10) adults. Values are means±SEMs. Blood glucose concentrations over time were analysed with 3-way repeated measures ANOVA (time × protein × age) and outcomes are reported in **Supplemental Table 1.** Blood glucose iAUC was analysed with 2-way ANOVA (protein × age) and outcomes are report in **Supplemental Table 2**. For iAUC data, young and older adults are displayed in the same panel as no age or age or protein × age effects were observed (both *P*>0.05) and Bonferroni *post hoc* tests were used to detect differences between protein sources (protein effects; both *P*<0.001). a, significant difference to CHLO (*P*<0.05); b, significant difference to MYCO (*P*<0.05); c, significant different to PEA (*P*<0.05); d, significant difference to SPIR (*P*<0.05). CHL, chlorella; iAUC, incremental area under the curve; LUP, lupin; MILK, milk; MYC, mycoprotein; PEA, pea; SPIR, spirulina.

**Supplemental Figure 3.** Hunger (A, B), satisfaction (C, D), fullness (E, F), prospective food intake (G, H), and composite appetite scores (I, J) during the 5 h postprandial period following the ingestion of 30 g protein from milk, mycoprotein, pea, lupin, spirulina, and chlorella in healthy young (*n*=12) and older (*n*=10) adults. Values are means±SEMs. Scores were analysed with 3-way repeated measures ANOVA (time × protein × age) and outcomes are reported in **Supplemental Table 6.** CHL, chlorella; LUP, lupin; MILK, milk; MYC, mycoprotein; PEA, pea; SPIR, spirulina.
