# Supplementary figures and images for: Ingestion of a variety of non-animal-derived dietary protein sources results in diverse postprandial plasma amino acid responses which differ between young and older adults
Source: Br J Nutr. 2024 Jan 15;131(9):1540–53. doi: 10.1017/S0007114524000163 (PMC11043913; doi:10.1017/S0007114524000163)

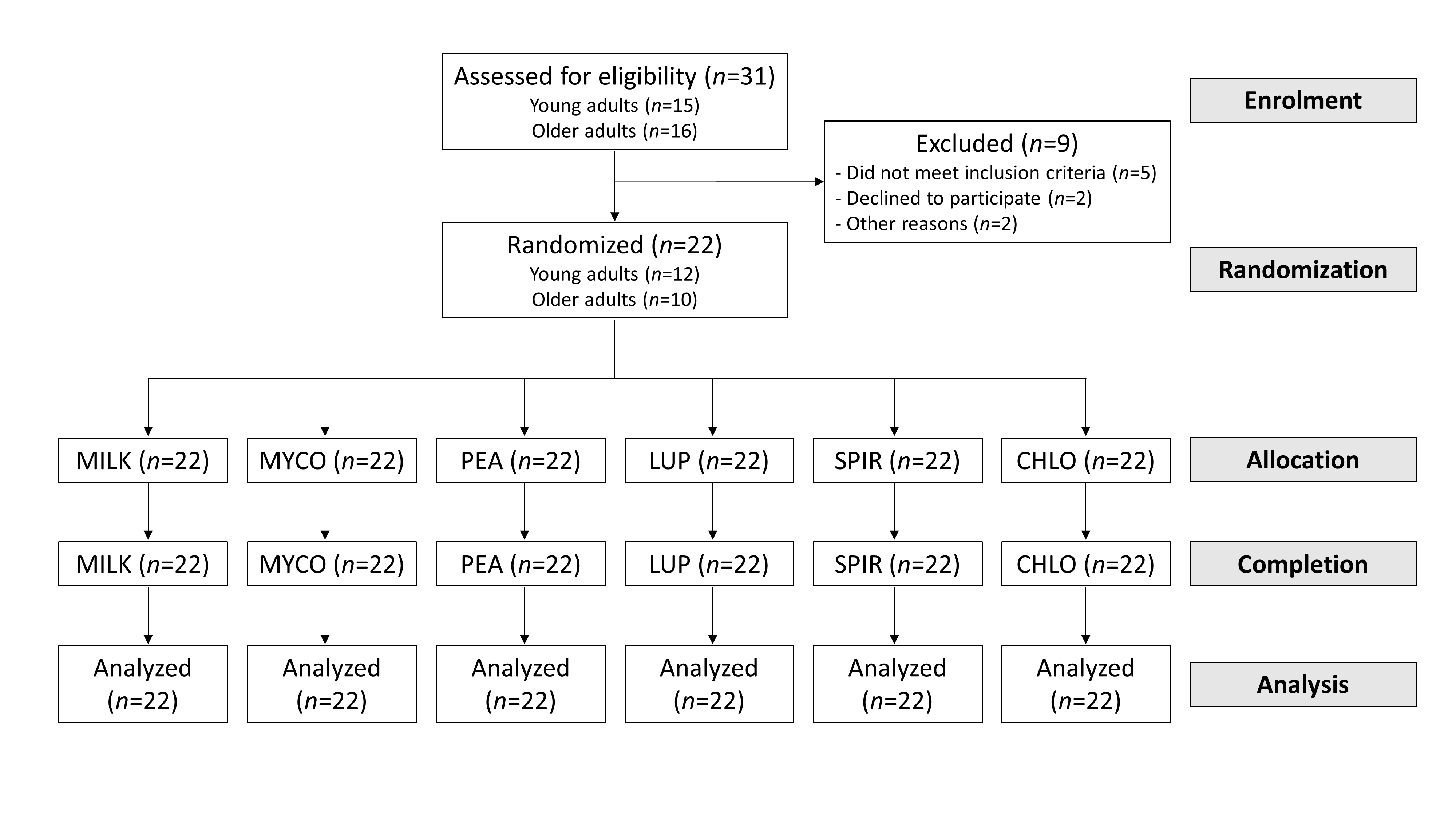

Supplement: van der Heijden et al. supplementary material 2 — van der Heijden et al. supplementary material [file S0007114524000163sup002.tif]

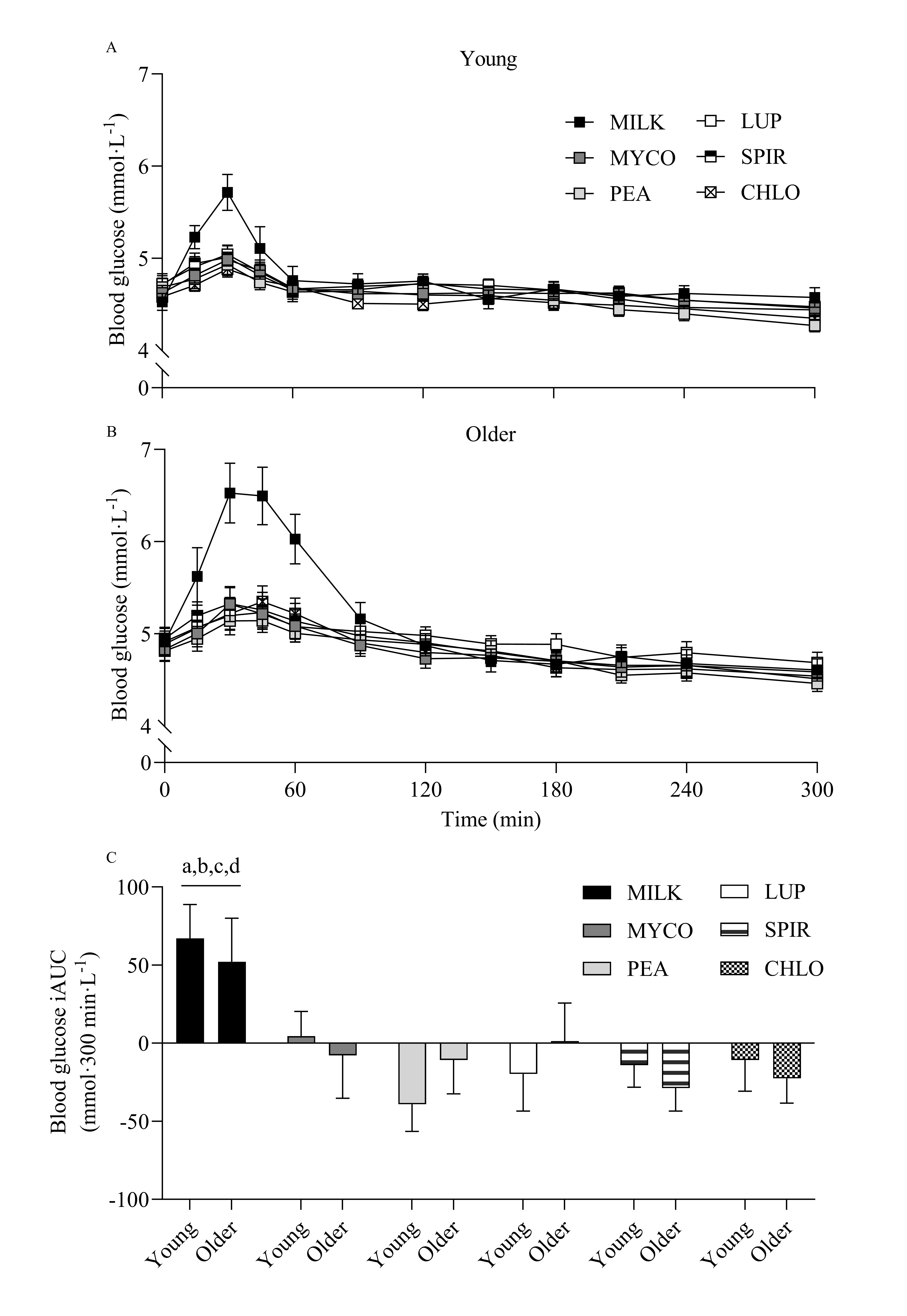

Supplement: van der Heijden et al. supplementary material 3 — van der Heijden et al. supplementary material [file S0007114524000163sup003.tif]

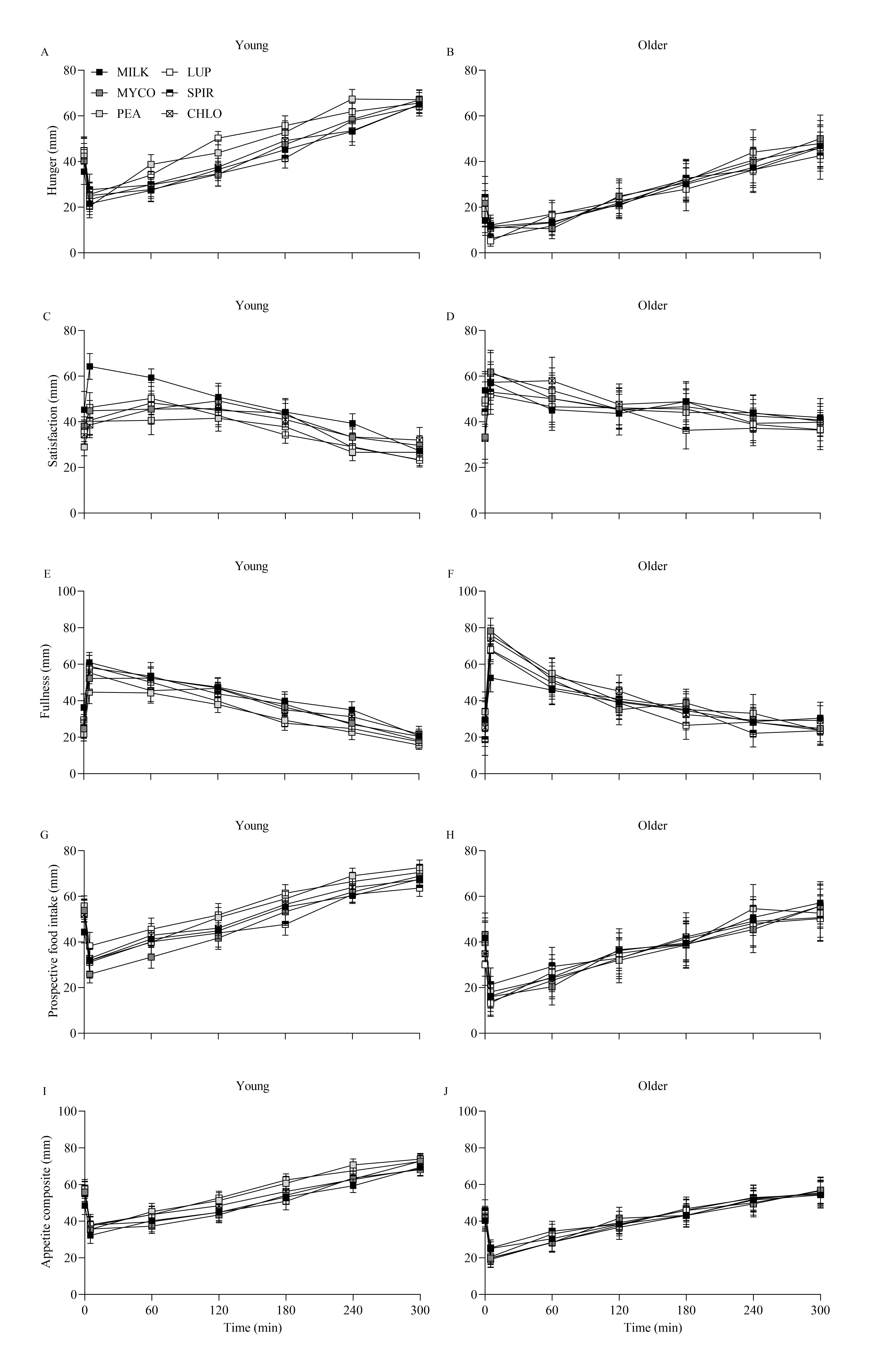

Supplement: van der Heijden et al. supplementary material 4 — van der Heijden et al. supplementary material [file S0007114524000163sup004.tif]
